# Mitochondrial genomes of eight Scelimeninae species (Orthoptera) and their phylogenetic implications within Tetrigoidea

**DOI:** 10.7717/peerj.10523

**Published:** 2021-02-02

**Authors:** Ran Li, Xiaoli Ying, Weian Deng, Wantao Rong, Xiaodong Li

**Affiliations:** 1 College of Life Sciences, Nanjing Normal University, Nanjing, China; 2 School of Chemistry and Bioengineering, Hechi University, Yizhou, China

**Keywords:** Tetrigoidea, Scelimeninae, Mitochondrial genome, Large intergenic spacer, Phylogenetic analysis

## Abstract

Scelimeninae is a key member of the pygmy grasshopper community, and an important ecological indicator. No mitochondrial genomes of Scelimeninae have been reported to date, and the monophyly of Scelimeninae and its phylogenetic relationship within Tetrigidae is still unclear. We sequenced and analyzed eight nearly complete mitochondrial genomes representing eight genera of Scelimeninae. These mitogenomes ranged in size from 13,112 to 16,380 bp and the order of tRNA genes between COII and ATP8 was reversed compared with the ancestral order of insects. The protein-coding genes (PCGs) of tetrigid species mainly with the typical ATN codons and most terminated with complete (TAA or TAG) stop codons. Analyses of pairwise genetic distances showed that ATP8 was the least conserved gene within Tetrigidae, while COI was the most conserved. The longest intergenic spacer (IGS) region in the mitogenomes was always found between tRNA^Ser(UCN)^ and ND1. Additionally, tandem repeat units were identified in the longest IGS of three mitogenomes. Maximum likelihood (ML) and Bayesian Inference (BI) analyses based on the two datasets supported the monophyly of Tetriginae. Scelimeninae was classified as a non-monophyletic subfamily.

## Introduction

Tetrigoidea (more than 2,000 species), the pygmy grasshoppers, is sister to Acridomorpha and belongs to the Acrididea infraorder (Song et al., 2015; Deng, 2016; Cigliano et al., 2020). The species in Tetrigoidea constitute a unique family, Tetrigidae, which is widely distributed throughout the world and has 265 genera within nine subfamilies (Scelimeninae, Metrodorinae, Diseotettiginae, Tetriginae, Cladonotinae, Lophotettiginae, Batrachideinae, Tripetalocerinae, and Cleostratinae) (Deng, 2016; Deng et al., 2019). Studies of Tetrigidae have mainly focused on their traditional morphological classification over the past decade due to its small size and marginal importance as an agricultural pest (Deng, 2016; Wei, Xin & Deng, 2018). The traditional subfamily Scelimeninae is one of the most diverse groups within Tetrigidae, and is composed of three main tribes (Scelimenini, Criotettigini and Thoradontini). Members of this subfamily generally inhabit moist environments, including wetlands and forest streams, and feed on lichens, mosses, and humus (Muhammad et al., 2018; Wei, Xin & Deng, 2018). They are regarded as important ecological indicators for the freshwater swamp forest because of their sensitivity to their microhabitat (Tan, Yeo & Hwang, 2017; Chen et al., 2018). However, the phylogenetic relationships between different genera of Scelimeninae are not well understood, nor is the taxonomic status of Scelimeninae within Tetrigidae (Deng, 2016; Adžić et al., 2020). The monophyly of Scelimeninae remains controversial (Muhammad et al., 2018; Adžić et al., 2020). Bolívar (1887) primarily defined Scelimeninae members based on the presence of lateral spines. However, Muhammad et al. (2018) and Adžić et al. (2020) proposed that Scelimeninae was probably not monophyletic. Previous studies determined that Tetriginae may have diverged more recently, based on several genes (COI, CYTB, 18S or 16S), while Scelimeninae is more basal (Fang et al., 2010; Jiang, 2000). Chen et al. (2018) analyzed the phylogenetic relationships among the genera of Scelimeninae with the combined dataset of COI, 16S, and 18S, however, their support values were relatively low. These results must be verified with better-defined molecular characteristics and larger sample size. Meanwhile, the sequences in that study are not publicly available, all the data still be regarded as speculative.

Insect mitochondrial genomes (mitogenomes) are generally double-strand circular molecules, 14–19 kb in size (Boore, 1999). They are composed of 13 protein-coding genes (PCGs), 22 transfer RNA genes (tRNAs), two ribosomal RNA genes (rRNAs), and a large non-coding region (called A + T-rich region) associated with the initiation of transcription and replication (Wolstenholme, 1992). The sequences of insect mitogenomes are good genomic makers for studies of molecular phylogenetics, population genetics, systematics, phylogeography, and molecular evolution because of unique features including the absence of introns, maternal inheritance, low rate of recombination, and high evolutionary rate (Saccone et al., 1999; Lin & Danforth, 2004; Simon et al., 2006; Avise, 2009; Krzywinski et al., 2011; Cameron, 2014; Qin et al., 2015). Complete mitogenomes have been shown to provide more highly supported results than single or multiple mitochondrial genes (Yuan et al., 2016; Liu et al., 2019). Until recently, only 18 mitogenome sequences of Tetrigidae have been submitted to the GenBank (https://www.ncbi.nlm.nih.gov/; last visited on July 30, 2020) (Xiao et al., 2012; Lin et al., 2017; Sun et al., 2017; Yang et al., 2017; Chang et al., 2020). Therefore, more mitogenomes of Tetrigidae are required for a comprehensive phylogenetic analysis, and before a full picture of the mitogenome for Tetrigidae can be depicted.

We sequenced and annotated eight mitogenomes of the traditional subfamily Scelimeninae. Furthermore, we analyzed the main features of the newly generated mitogenomes and those of other tetrigid species. We analyzed the monophyly of Scelimeninae and its phylogenetic relationship within Tetrigidae for the first time at the mitogenome level. Our results may improve our understanding of the genomics, phylogenetics, and evolution of the family Tetrigidae.

## Materials and Methods

### Taxon sampling and DNA extraction

The samples analyzed in this study are shown in Table S1. All newly sequenced samples (eight adults of Scelimeninae species) were collected from Hechi, Luocheng, Huanjiang, Shangsi, Rongshui, Yizhou of Guangxi Province, and Ya-an of Sichuan Province, China, respectively. All specimens were morphologically identified by Dr. Weian Deng using available taxonomic keys. Total genomic DNA was extracted from the hind femoral muscles of each specimen using a Wizard^®^ Genomic DNA Purification Kit (Promega, Madison, WI, USA) according to the manufacturer’s instructions. The quality of total DNA was checked with 1% agarose gel and the concentration was measured with a Nanodrop 2000 spectrophotometer (Thermo, Wilmington, NC, USA). Voucher specimens and DNA samples were subsequently preserved at −80 °C and deposited in the Museum of Insects of Hechi University, Guangxi, China. We did not require a permit for our study.

### Primer design, PCR amplification and sequencing

The mitogenomes of all species were generated by amplifying overlapping fragments using a specific set of certain pairs of universal primers for grasshopper mitogenomes (Simon et al., 1994, 2006) and newly-designed specific primers for Tetrigidae mitogenomes (Table 1). All PCR amplifications were executed using MyCycler Thermal Cycler (Bio-Rad, Hercules, CA, USA). The procedures were performed using *Ex*-Taq polymerase (Takara, Dalian, China), following the conditions of our previous study (Li et al., 2020). The size of PCR products was determined using 1% agarose gel with TAE buffer, and they were sequenced with the ABI 3730XL DNA Analyzer by Genscript Biotech Corp. (Nanjing, China) in both forward and reverse directions.

**Table 1:** Universal and specific primers for Scelimeninae mitogenomes used in this study.

No.	Primers (5′-3′)
TW-J-1310	GTTAATAAAACTAATAACCTTCAAA
C1-N-2776	GATAATCAGAATATCGTCGTGG
N5-J-6579	CTCACCTCAACCAGAATC
N4-N-8484	TCTAATATGGCTTCTCCTCC
CB-J-11545	ACATGAATTGGAAAACGACCAGT
LR-N-12866	ACATGATCTGAGTTCAGACCGG
TF-J-6400	TAACATCTTCAATGTTATACTCT
N5-N-7211	TTAAGGCTTTATTATTCATGTGTGC
N5-J-7572	AAAAGGAATTTGTGCTCTCTTAGT
N4-N-8727	AAAAGGATTATTGCTTATTCTTC
TK-J-3790	CATCAGATGACTGAAAGTAAGTA
N3-N-5731	TTAGGGTCAAATCCACATTC
CB-J-10933	GTTTTACCATGAGGTCAAATATC
CB-N-11526	TTCTACTGGTCGTTTTCCAATTCA
LR-J-13900	TGATAAACCCTGATACAAAAG
SR-N-14745	GTGCCAGCAGCCGCGGTTATAC
TF-J-34	GCCTGATAAAAAGGRTTAYYTTGATA
TR-N-1284	ACARCTTTGAAGGYTAWTAGTTT
16S-F	CGCCTGTTTATCAAAAACAT
16S-R	CTCCGGTTTGAACTCAGATCA
T-F-2899	ACAATTGGTCAYCAATGATAYTG
T-R-4052	ATGTCCWGCAATYATATTWGC
TF-9148	ACCTAAAGCTCCCTCACAWAC
TR-10510	TATCTACAGCRAATCCYCCYCA

### Mitochondrial genome assembly, annotation and analysis

All sequences were checked and assembled using the SeqMan program from the Lasergene software package (DNASTAR, Madison, WI, USA). Eight assembled mitogenomes were firstly uploaded to the MITOS web server (http://mitos2.bioinf.uni-leipzig.de/) for initial annotation under the code for invertebrate mitochondria (Bernt et al., 2013). However, the MITOS web server did not correctly identify the start and stop codons, so all PCGs were further adjusted and corrected manually using the reference mitogenomes of *Tetrix japonica* (Bolívar, 1887) and *Alulatettix yunnanensis* (Liang, 1993; Xiao et al., 2012; Sun et al., 2017). rRNA genes were identified by alignment with homologous genes of previously sequenced mitogenomes from the family Tetrigidae. Intergenic spacers and overlapping regions between genes were estimated manually. The tandem repeats (position, number, and length) in the control region were predicted using Tandem Repeat Finder 4.07 (http://tandem.bu.edu/trf/trf.html) with default settings (Benson, 1999). Base composition, codon distribution, relative synonymous codon usage (RSCU) of PCGs was calculated in MEGA 7 (Kumar, Stecher & Tamura, 2016). Nucleotide compositional differences (composition skew) was measured based on the formula: AT-skew [(A − T)/(A + T)] and GC-skew [(G − C)/(G + C)] (Perna & Kocher, 1995). The genetic distances for different PCGs among the Tetrigidae species were estimated using MEGA 7 (Kumar, Stecher & Tamura, 2016).

### Sequence alignment and phylogenetic inference

A total of 22 mitogenomes were used for phylogenetic analyses, including eight species sequenced in this study, 12 additional species of Tetrigidae retrieved from GenBank, and two species of Tridactyloidea (*Mirhipipteryx andensis* Günther, 1969 and *Ellipes minuta* (Scudder, 1862)) used as outgroups. Sequences for each PCG were aligned individually with codon-based multiple alignments using MAFFT version 7.205 by codon using the G-INS-I algorithm (Katoh et al., 2002; Li et al., 2016). Nucleotide saturation was tested in DAMBE 5 (Xia & Xie, 2001). The program Gblocks 0.91b was used with default settings to identify the poorly aligned positions and divergent regions (Castresana, 2000). The individual alignment fragments were then concatenated by FASconCAT-G version 1.0 (Kück & Longo, 2014).

Phylogenetic analyses were conducted using two datasets (PCG123 and PCG12, concatenated alignments of PCGs with and without third codon positions) by Bayesian inference (BI) and maximum likelihood (ML) methods. The optimal partitioning strategy and best-fitting model were estimated by the program PartitionFinder 1.1.1 (Lanfear et al., 2012) under the Bayesian information criterion (BIC) (Tables 2 and 3). ML analyses were conducted in RAxML through the online CIPRES Science gateway, and the best tree was calculated with branch support estimated 1000 bootstrap replicates (Miller, Pfeiffer & Schwartz, 2010; Liu, Linder & Warnow, 2011; Stamatakis, 2014). BI analyses were performed in MrBayes 3.2.6, and two simultaneous runs with four chains (three heated and one cold) were performed for 10 million generations and were sampled every 1,000 trees (Ronquist & Huelsenbeck, 2003). The initial 25% of trees from each run was discarded as burn-in and the consensus tree was computed from the remaining trees. The phylogenetic trees are visualized in FigTree 1.4.2 (Rambaut, 2019).

**Table 2:** The partition schemes and best-fitting models selected in PCG123 dataset.

Subset	Nucleotide sequence alignment	
	Partition name	Best model
Partition 1	ND6_pos1, ATP8_pos1, ND3_pos1, ATP6_pos1, ND2_pos1	GTR+I+G
Partition 2	COI_pos2, COIII_pos2, COII_pos2, ATP6_pos2, CYTB_pos2	GTR+I+G
Partition 3	ND3_pos3, CYTB_pos3, COI_pos3, COII_pos3, COIII_pos3, ATP6_pos3	HKY+G
Partition 4	ND6_pos2, ATP8_pos2, ND3_pos2, ND2_pos2	GTR+G
Partition 5	ND6_pos3, ND2_pos3, ATP8_pos3	HKY+G
Partition 6	COI_pos1, COIII_pos1, COII_pos1, CYTB_pos1	GTR+I+G
Partition 7	ND1_pos1, ND4L_pos1, ND5_pos1, ND4_pos1	GTR+I+G
Partition 8	ND5_pos2, ND4L_pos2, ND4_pos2, ND1_pos2	GTR+I+G
Partition 9	ND1_pos3, ND5_pos3, ND4_pos3, ND4L_pos3	GTR+G

**Table 3:** The partition schemes and best-fitting models selected in PCG12 dataset.

Subset	Nucleotide sequence alignment	
	Partition name	Best model
Partition 1	ND6_pos1, ATP8_pos1, ND3_pos1, ATP6_pos1, ND2_pos1	GTR+I+G
Partition 2	COIII_pos2, COI_pos2, COII_pos2, ATP6_pos2, CYTB_pos2	GTR+I+G
Partition 3	ND6_pos2, ATP8_pos2, ND2_pos2, ND3_pos2	GTR+G
Partition 4	COI_pos1, COIII_pos1, CYTB_pos1, COII_pos1	GTR+I+G
Partition 5	ND1_pos1, ND4L_pos1, ND4_pos1, ND5_pos1	GTR+I+G
Partition 6	ND5_pos2, ND4L_pos2, ND1_pos2, ND4_pos2	GTR+I+G

## Results and discussion

### Mitogenome structure, organization

**Figure 1:**
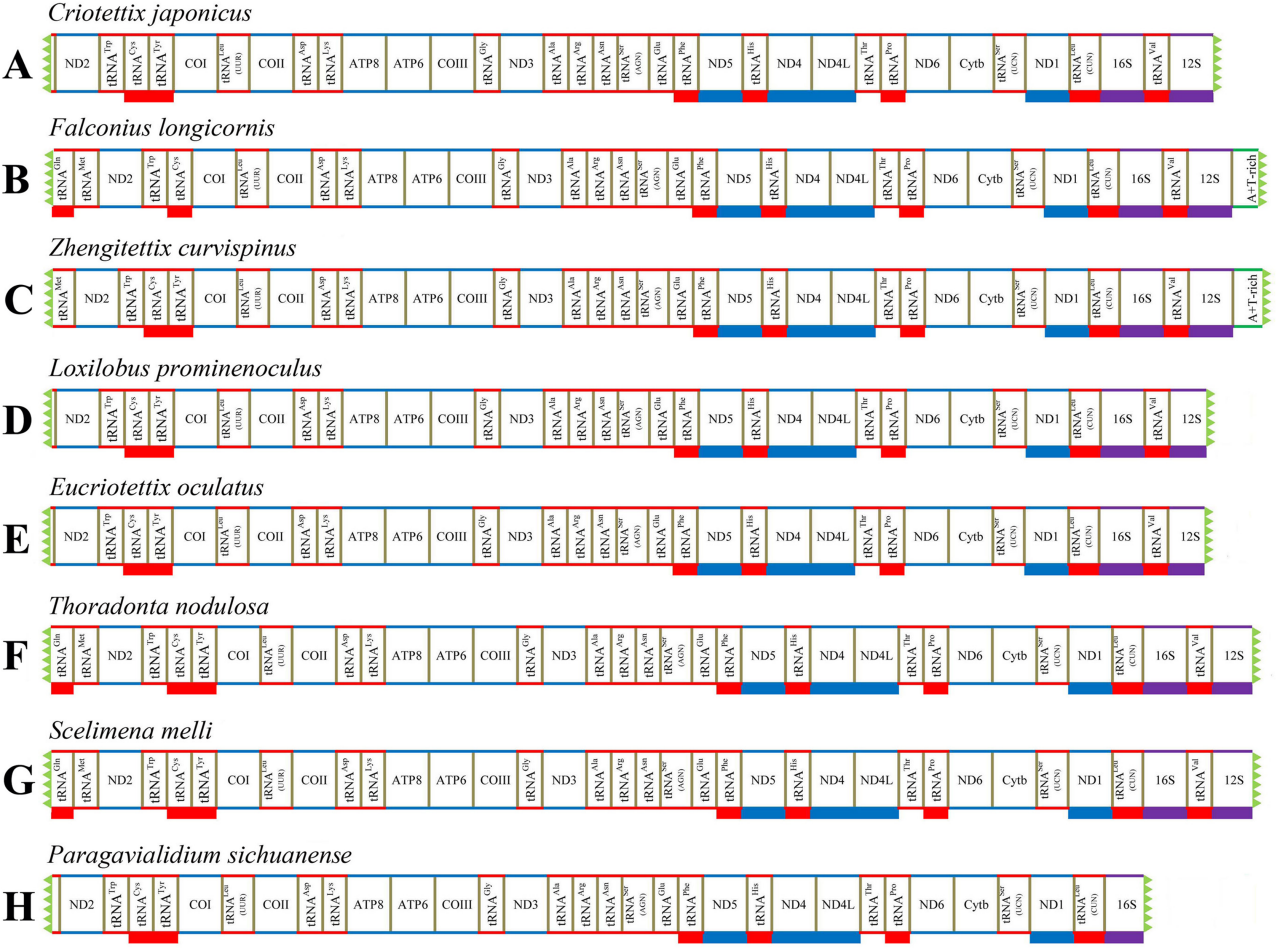
Mitogenome structure and organization of Scelimeninae. (A) *Criotettix japonicus*, (B) *Falconius longicornis*, (C) *Zhengitettix curvispinus*, (D) *Loxilobus prominenoculus*, (E) *Eucriotettix oculatus*, (F) *Thoradonta nodulosa*, (G) *Scelimena melli*, (H) *Paragavialidium sichuanensis*.

We successfully obtained the mitogenomes of eight species of the subfamily Scelimeninae (*Criotettix japonicus* (Haan, 1843); *Falconius longicornis* Deng, Zheng & Wei, 2009; *Zhengitettix curvispinus* Liang, Jiang & Liu, 2007; *Loxilobus prominenoculus* Zheng & Li, 2001; *Eucriotettix oculatus* (Bolívar, 1898); *Thoradonta nodulosa* (Stål, 1861); *Scelimena melli* Günther, 1938; *Paragavialidium sichuanense* Zheng, Wang & Shi, 2007). The newly sequenced mitogenomes were submitted to GenBank (accession numbers: MT162542–MT162549). All sequences were nearly complete mitogenomes, from 13,112 bp in *P. sichuanense* to 16,380 bp in *Z. curvispinus* (Tables S2–S9). We failed to sequence regions found between tRNA^Val^ and ND2 that generally contained 12S (partial), tRNA^Ile^, tRNA^Gln^, tRNA^Met^ and a putative A + T-rich region. The gene tRNA^Val^ was also not obtained for *P. sichuanense*, which resulted in the smallest sequence (Fig. 1). The gene arrangement of the mitogenomes was identical to that of other Acrididea species (Tetrigoidea and Acridomorpha) (Fig. 1). The order of the tRNA genes between COII and ATP8 was reversed, which was opposite to the ancestral insect arrangement (*Drosophila yakuba*) in which tRNA^Lys^ preceded tRNA^Asp^ (Clary & Wolstenholme, 1985). Our results support the hypothesis that this rearrangement has persisted for nearly 250 million years since Acridoidea diverged from Tridactylidea (Song et al., 2015). As a consequence, this pattern of gene arrangement can be regarded as a clear molecular synapomorphy for Acrididea.

### Protein-coding genes

A total of 13 protein-coding genes were detected in all newly sequenced mitogenomes. Similar to other Acrididea mitogenomes, nine PCGs were encoded on the majority strand (J-strand), and the remaining four PCGs (ND1, ND4, ND4L and ND5) were encoded on the minority strand (N-strand) (Fig. 1). We studied the nucleotide composition of mitogenome using the general parameters, including A + T content, AT-skew, and GC-skew (Li et al., 2020). Comprehensive analysis of all PCGs of the eight Scelimeninae species and other available tetrigid species showed that they possessed highly similar nucleotide composition biases towards A and T nucleotides, ranging from 66.65% (*S. melli*) to 75.15% (*Systolederus spicupennis*) on the J-strand (Table 4), which was identical to most orthopteran species. Our comparative analysis also revealed a different total length (11,026–11,150 bp), however, two pairs of species had the same length (Table 4). The skew metrics of the PCGs within all tetrigid species showed a negative AT-skew and a negative GC-skew (except for *C. japonicus*, *S. spicupennis* and *T. japonica*), indicating that Ts and Cs were more abundant than As and Gs in most tetrigid mitogenomes (Table 4).

**Table 4:** Composition and skewness of PCGs of the studied tetrigid mitogenomes.

Species	Total (bp)	A+T (%)	AT-skew	GC-skew
*Criotettix japonicus*	11,090	75.07	−0.1255	0.0105
*Falconius longicornis*	11,122	68.76	−0.1186	−0.0492
*Zhengitettix curvispinus*	11,150	74.80	−0.1132	−0.0206
*Loxilobus prominenoculus*	11,112	72.24	−0.1220	−0.0528
*Eucriotettix oculatus*	11,115	74.10	−0.1202	−0.0379
*Thoradonta nodulosa*	11,112	70.52	−0.1246	−0.0525
*Scelimena melli*	11,135	66.65	−0.1195	−0.0539
*Paragavialidium sichuanense*	11,126	71.27	−0.1402	−0.0213
*Trachytettix bufo*	11,123	70.61	−0.1085	−0.0572
*Yunnantettix bannaensis*	11,096	69.62	−0.1120	−0.0525
*Bolivaritettix lativertex*	11,126	72.75	−0.1231	−0.0238
*Systolederus spicupennis*	11,026	75.15	−0.1178	0.0234
*Alulatettix yunnanensis*	11,124	74.51	−0.1077	−0.0102
*Coptotettix longjiangensis*	11,104	72.08	−0.1102	−0.0200
*Formosatettix qinlingensis*	11,098	74.76	−0.1084	−0.0046
*Ergatettix dorsifera*	11,106	70.58	−0.1111	−0.0291
*Euparatettix bimaculatus*	11,085	71.82	−0.1134	−0.0339
*Euparatettix variabilis*	11,085	72.51	−0.1145	−0.0299
*Tetrix ruyuanensis*	11,094	74.76	−0.1090	−0.0007
*Tetrix japonica*	11,110	74.79	−0.1087	0.0011

A comprehensive analysis of predicted initiation codons showed that the PCGs of tetrigid species mainly started with a typical ATN codon (ATG, ATA, ATC, and ATT) (Table 5; Table S10). Seven genes were conserved among all species as well as the initiation codons ATG (for ATP6, ATP8, COIII, CYTB and ND4), ATC (for COI) and ATT (ND4L). Several other start codons were discovered, including COII of *T. bufo* with the initiation codon GTG, and ND5 of *P. sichuanense* with the initiation codon TTG (Table 5; Table S10). For the termination codons, all PCGs of tetrigid species terminated with complete (TAA or TAG) or truncated (T) stop codons. The genes ATP6, ATP8, COII, ND4L, and ND6 ended with the complete codon TAA. ND2 of all mitogenomes (except for *A. yunnanensis*) ended with TAA. In addition, COIII and ND5 almost terminated with T, which is a common phenomenon in the mitogenomes of metazoans and can produce functional terminal codons via polycistronic transcription cleavage and polyadenylation processes (Ojala, Montoya & Attardi, 1981).

**Table 5:** Non-conservative start and stop codons of PCGs in tetrigid mitogenomes.

Species	Gene								
	COI	COIII	CYTB	ND1	ND2	ND3	ND4	ND5	ND6
*C. japonicus*	ATC/TAA	ATG/TAA	ATG/TAA	ATA/TAA	ATT/TAA	GTA/TAA	ATG/TAG	ATG/T	TTG/TAA
*F. longicornis*	ATC/TAA	ATG/TAA	ATG/TAG	ATT/TAA	ATA/TAA	ATA/TAG	ATG/TAG	ATG/T	ATG/TAA
*Z. curvispinus*	ATC/TAA	ATG/T	ATG/TAA	ATT/TAA	ATT/TAA	ATA/TAG	ATG/TAA	ATG/T	ATC/TAA
*L. prominenoculus*	ATC/TAA	ATG/TAA	ATG/TAA	ATA/TAA	ATC/TAA	ATC/TAG	ATG/TAG	ATG/TAA	TTG/TAA
*E. oculatus*	ATC/TAA	ATG/TAG	ATG/TAA	ATA/TAA	ATC/TAA	ATT/TAG	ATG/TAA	ATG/TAA	TTG/TAA
*T. nodulosa*	ATC/TAG	ATG/TAA	ATG/TAA	ATT/TAA	ATT/TAA	ATA/TAG	ATG/TAG	ATG/TAA	TTG/TAA
*S. melli*	ATC/TAA	ATG/T	ATG/TAG	ATA/TAA	ATC/TAA	ATT/TAG	ATG/TAA	ATG/T	TTG/TAA
*P. sichuanense*	ATC/TAA	ATG/T	ATG/TAG	ATT/TAA	ATT/TAA	ATT/TAG	ATG/TAG	TTG/T	ATG/TAA
*T. bufo*	ATC/T	ATG/T	ATG/TAG	ATT/TAG	ATG/TAA	ATT/TAG	ATG/TAG	ATG/T	TTG/TAA
*Y. bannaensis*	ATC/T	ATG/TAA	ATG/TAG	ATT/TAA	ATT/TAA	ATT/TAG	ATG/TAG	ATG/T	TTG/TAA
*B. lativertex*	ATC/T	ATG/TAG	ATG/TAA	ATT/TAA	ATT/TAA	ATT/TAG	ATG/TAG	ATG/T	TTG/TAA
*S. spicupennis*	ATC/T	ATG/T	ATG/T	ATT/TAA	ATG/TAA	ATT/TAG	ATG/TAG	ATG/T	ATT/TAA
*A. yunnanensis*	ATC/TAA	ATG/TAA	ATG/T	ATA/TAA	ATG/T	ATA/TAG	ATG/T	ATG/T	ATG/TAA
*C. longjiangensis*	ATC/T	ATG/T	ATG/TAA	ATT/TAA	GTG/TAA	ATA/T	ATG/TAG	ATG/T	ATG/TAA
*F. qinlingensis*	ATC/T	ATG/T	ATG/TAG	ATT/TAA	ATG/TAA	ATT/T	ATG/TAG	ATG/T	ATG/TAA
*E. dorsifera*	ATC/T	ATG/T	ATG/TAG	ATT/TAG	ATG/TAA	ATC/TAG	ATG/TAG	ATG/T	ATT/TAA
*E. bimaculatus*	ATC/T	ATG/T	ATG/TAG	ATT/TAA	GTG/TAA	ATC/TAG	ATG/TAG	ATG/T	ATT/TAA
*E. variabilis*	ATC/T	ATG/T	ATG/TAG	ATT/TAA	GTG/TAA	ATT/TAG	ATG/TAG	ATG/T	ATG/TAA
*T. ruyuanensis*	ATC/T	ATG/T	ATG/TAG	ATT/TAA	ATG/TAA	ATC/TAG	ATG/TAG	ATG/T	ATG/TAA
*T. japonica*	ATC/TAA	ATG/T	ATG/TAG	ATA/TAA	ATG/TAA	ATA/TAG	ATG/TAG	ATG/TAA	ATG/TAA

**Figure 2:**
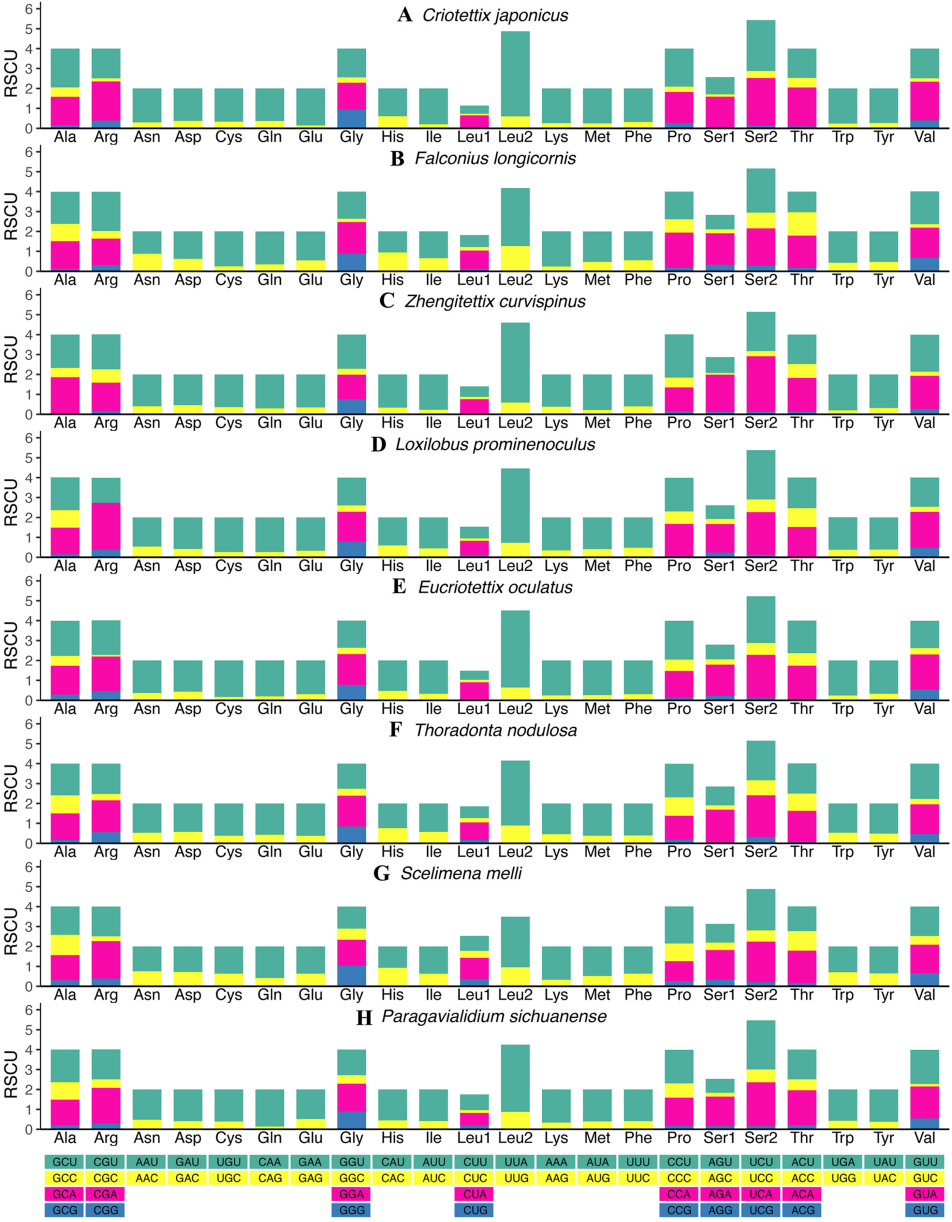
The relative synonymous codon usage (RSCU) in the mitogenomes of Scelimeninae. (A) *Criotettix japonicus*, (B) *Falconius longicornis*, (C) *Zhengitettix curvispinus*, (D) *Loxilobus prominenoculus*, (E) *Eucriotettix oculatus*, (F) *Thoradonta nodulosa*, (G) *Scelimena melli*, (H) *Paragavialidium sichuanensis*.

The relative synonymous codon usage (RSCU) values of all new mitogenomes were calculated and summarized in Fig. 2 and Tables S11–S18. Apart from the termination codons, the total number of codons of the eight mitogenomes was similar, ranging from 3,684 (*C. japonicus*) to 3,700 (*S. melli*). Comparative analysis showed that the codon usage patterns and the major customarily utilized codons of the eight mitogenomes were conserved. Codon usage analysis showed that the most frequently used codons were TTT, ATT, UUA, AUA. Meanwhile, the most frequently used amino acids were Leucine 2 (Leu 2), Isoleucine (Ile) and Phenylalanine (Phe). Similarly, the biased use of A + T nucleotides was reflected in the codon frequencies. RSCU analysis also indicated that codons used more A + T at the third codons (Fig. 2).

**Figure 3:**
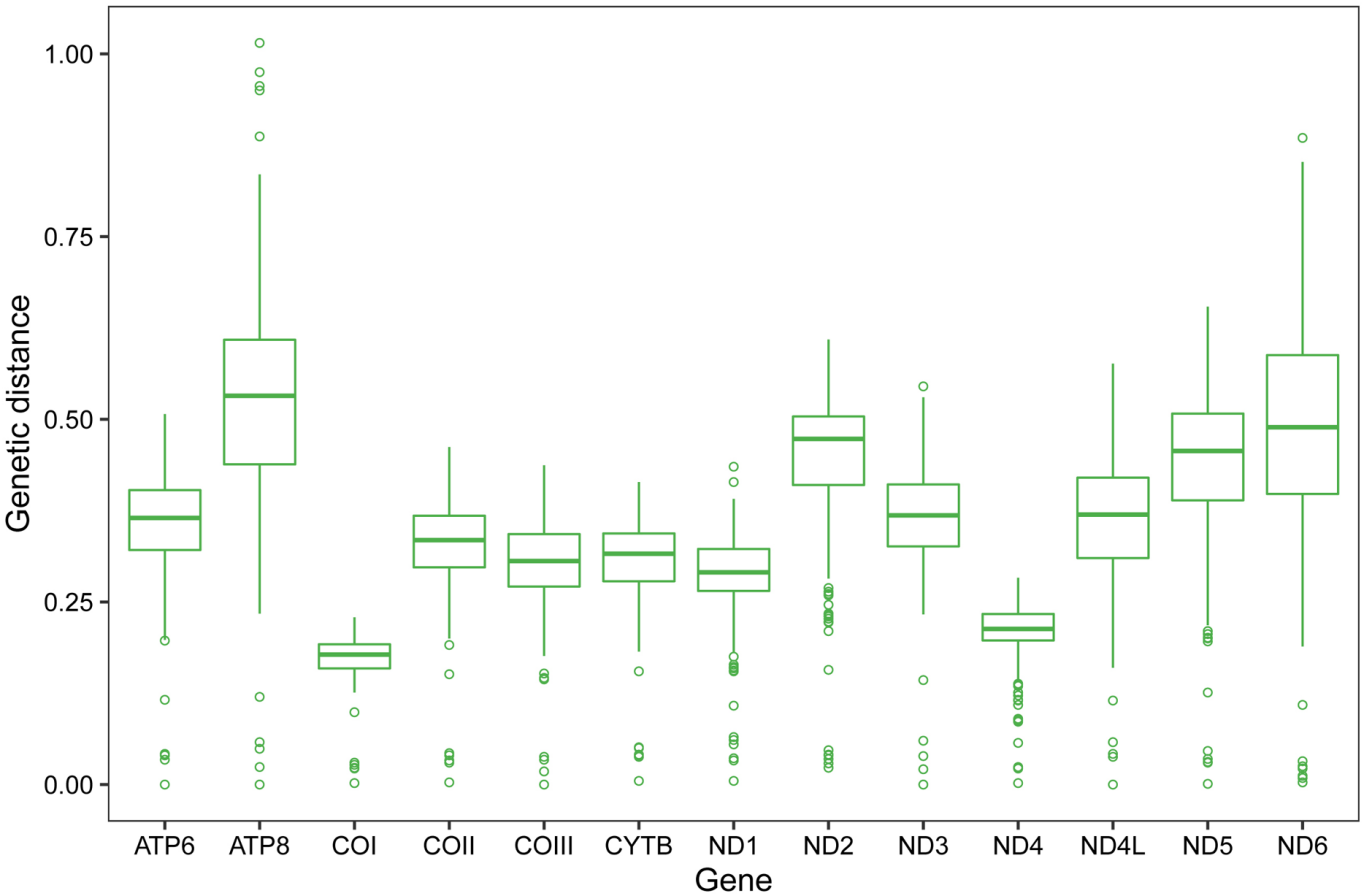
Genetic distances of individual protein-coding gene among 20 mitogenomes within Tetrigidae.

Pairwise genetic distances of single PCG among the eight Scelimeninae and other 12 valid mitogenomes within Tetrigidae are summarized in Fig. 3. COI was the most conserved gene (average 0.1723, range 0.0024–0.2285), and ATP8 was the least conserved (average 0.5240, range 0.0241–1.0149). This is a trait in the mitogenomes of metazoans and is evidence that COI is one of the most useful molecular markers for inferring phylogenetic relationships in Tetrigidae (Suh et al., 2019).

### Transfer and ribosomal RNA genes

Owing to the nearly complete mitogenomes, 22 typical tRNAs which generally occurred in Acrididea species were not all recognized in the eight Scelimeninae species. A total of 20 tRNAs were obtained in four mitogenomes (*F. longicornis*, *Z. curvispinus*, *T. nodulosa*, and *S. melli*), 19 in three mitogenomes (*C. japonicus*, *L. prominenoculus* and *E. oculatus*), and 18 in *P. sichuanense* (Fig. 1; Tables S2–S9). Notably, the gene of tRNA^Tyr^ was not found between tRNA^Cys^ and COI in the genome of *F. longicornis*, which is not consistent with other available mitogenomes of Acrididea species. For this unexpected rearrangement, we ruled out the mistake of annotation, and the mechanism for this arrangement should be explored in future studies. All tRNAs obtained were interspersed in the mitogenomes and ranged from 58 bp to 73 bp, with the shortest being the tRNA^Trp^ of *C. japonicus* and the tRNA^Val^ of *Z. curvispinus* was the longest.

Two rRNA genes (16S rRNA and 12S rRNA) were generally observed in all available Acrididea complete mitogenomes, and the 16S gene was located between tRNA^Leu(CUN)^ and tRNA^Val^, while 12S gene was between tRNA^Val^ and the control region. The complete 16S rRNA genes were identified in seven Scelimeninae mitogenomes, with sizes ranging from 1,277 bp for *E. oculatus* to 1,295 bp for *S. melli*. However, the partial gene was sequenced in *P. sichuanense* with 850 bp long. Only two complete sequences were obtained in *F. longicornis* (778 bp) and *Z. curvispinus* (781 bp) for the 12S gene.

### Non-coding regions

**Figure 4:**
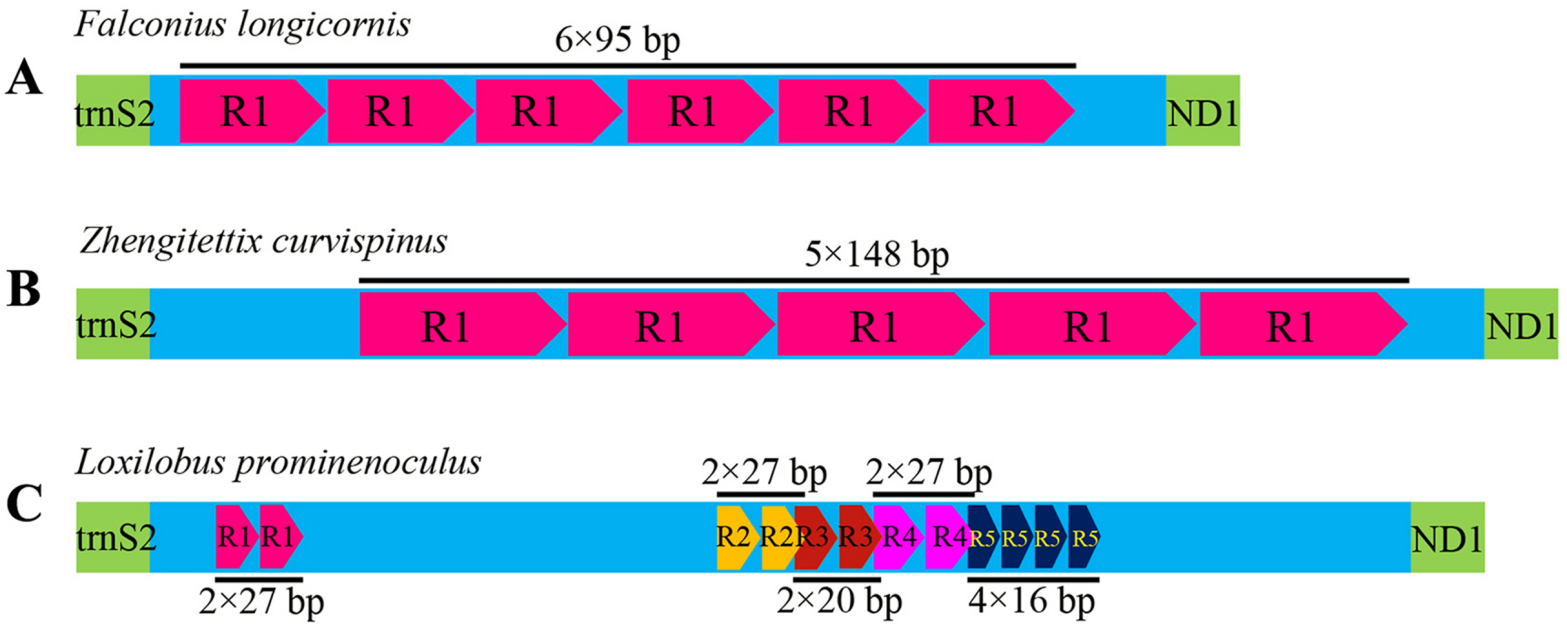
The putative structures in the longest intergenic spacer (IGS) region of *F. longicornis* (A), *Z. curvispinus* (B) and *L. prominenoculus* (C).

Like most insects, the different length of mitogenomes among Scelimeninae is mainly due to the size variation of the non-coding regions (Lv, Li & Kong, 2018). Comparative analysis of the sequences showed that the number and size of the overlapping regions and intergenic spacer regions between species was significantly different, which was inconsistent with other Acrididea mitogenomes. There were 13–17 overlapping regions varying from 1 bp to 12 bp in size within the eight Scelimeninae mitogenomes. Interestingly, the gene overlaps presented at the boundary of ATP6/ATP8 and ND4/ND4L were conserved in all 20 valid mitogenomes of Tetrigidae. The 7 bp nucleotide sequences (5′-ATGATAA-3′ and 3′-ATGTTAA-5′) were identical to those of all analyzed mitogenomes, which is commonly observed in many other Acrididea mitogenomes. A total of 7–11 intergenic regions with larger differences in size were present in the eight new mitogenomes. The longest intergenic spacer (IGS) region in the mitogenomes was found between tRNA^Ser(UCN)^ and ND1, ranging from 11 bp to 945 bp in size. *S. melli* had 72 nucleotides between the tRNA^Ala^ and tRNA^Arg^ genes (Tables S2–S9). Notably, the IGS of *F. longicornis* possessed six copies of tandem repeats with a fragment of 95 bp (Fig. 4A), and the sequence of *Z. curvispinus* also contained one tandem repeat of a 148 bp fragment (Fig. 4B). In addition, the region of *L. prominenoculus* possessed five tandem repetitive sequences: R1 (2 × 27 bp), R2 (2 × 27 bp), R3 (2 × 20 bp), R4 (2 × 27 bp) and R5 (4 × 16 bp) (Fig. 4C). The existence of tandem repeats of orthopteran mitogenomes has been generally observed in the control region, however, it was first found in the intergenic spacer region. Therefore, more tetrigid mitogenomes are needed to understand the pattern and mechanism of evolution.

### Phylogenetic analysis

We used two datasets to conduct phylogenetic analyses, which included or excluded specific sites. The PCG123 matrix consisted of 10,722 sites and included all codon positions of the 13 PCGs. The PCG12 matrix contained 7,148 sites, including only the first and second codon positions of 13 PCGs. DAMBE analysis showed a lower ISS value than the ISS.c (*P* ≤ 0.0001), which confirmed the suitability of the nucleotide’s unsaturation of the two datasets and the sequences for phylogenetic analyses. We constructed Maximum Likelihood (ML) and Bayesian Inference (BI) trees of 20 species from four subfamilies (Tetriginae, Metrodorinae, Cladonotinae and Scelimennae) of Tetrigidae and two species (outgroups) with best partition schemes.

**Figure 5:**
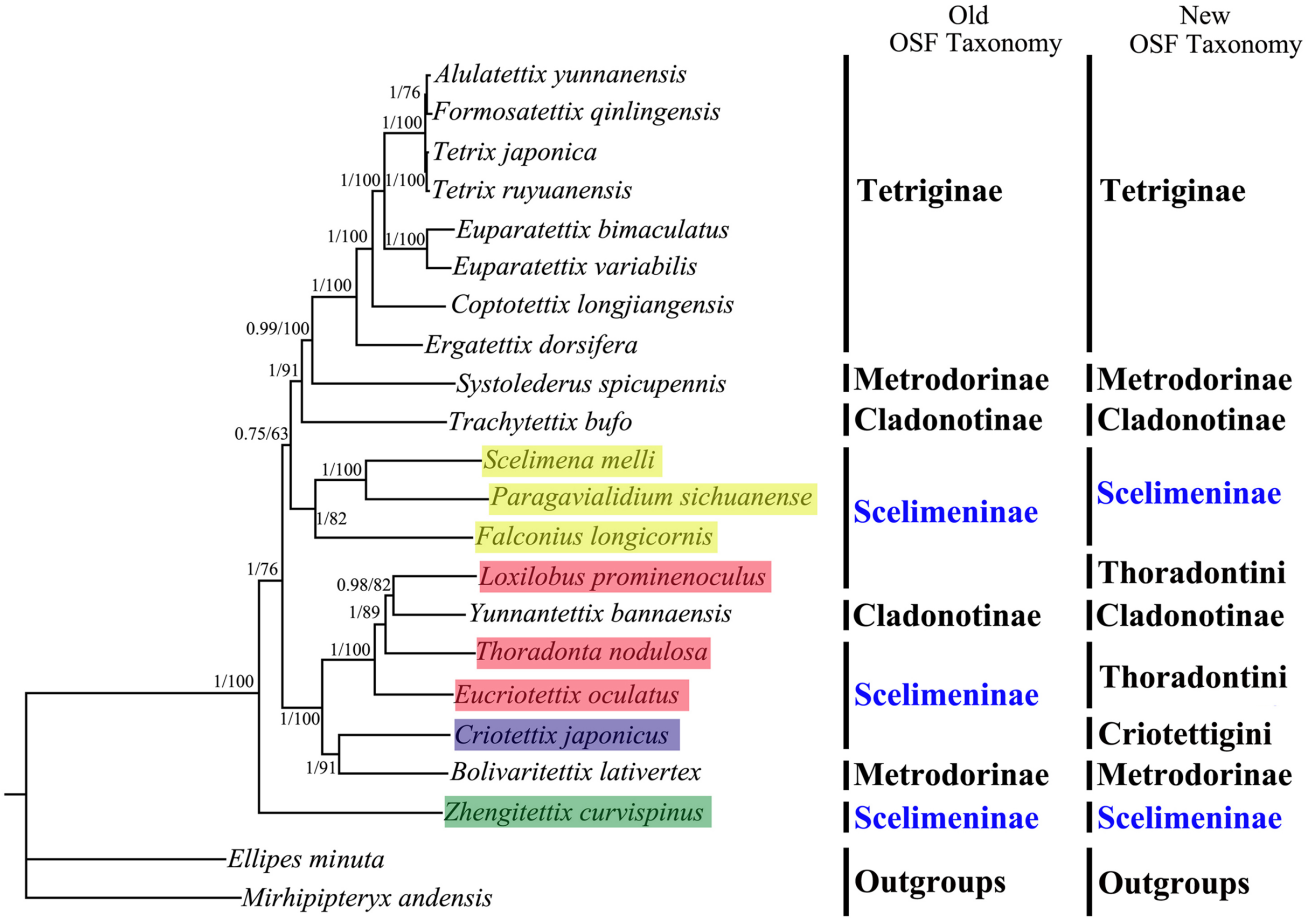
A phylogenetic tree obtained from BI and ML analysis based on the PCG123 dataset. The numbers at the nodes separated by “/” indicate the posterior probability (BI) and bootstrap value (ML).

**Figure 6:**
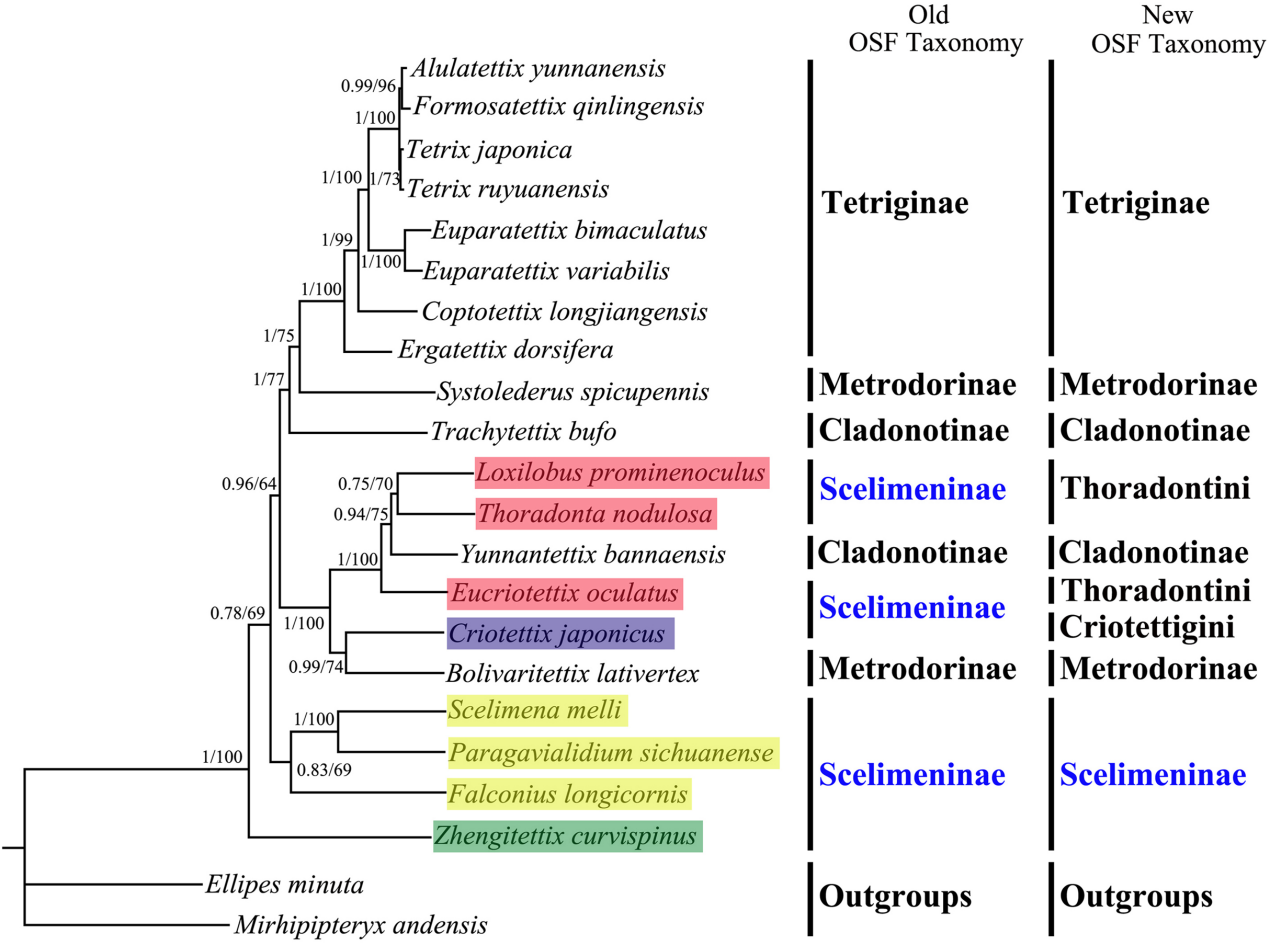
A phylogenetic tree obtained from BI and ML analysis based on the PCG12 dataset. The numbers at the nodes separated by “/” indicate the posterior probability (BI) and bootstrap value (ML).

All phylogenetic analyses using the same data matrices yet different methods yielded the same topology (Figs. 5 and 6). Tetriginae was monophyletic in all analyses with current species and the subfamily was the more recently diverged group. This was also supported by previous phylogenetic studies (Jiang, 2000; Fang et al., 2010; Zhang et al., 2020). In addition, the results from four trees showed that *S. spicupennis* of Metrodorinae clustered near the clade Tetriginae, *Bolivaritettix lativertex* was closely related to *C. japonicus* of Scelimeninae. All trees showed *Trachytetix bufo* and *Yunnantettix bannaensis* did not form one sister group for Cladonotinae. Our results supported the previous finding that Metrodorinae and Cladonotinae were not monophyletic groups (Yao, 2008; Pavon-Gozalo, Manzanilla & Garcia-Paris, 2012; Lin et al., 2015). As only two species were included in the subfamilies, more samples are needed to uncover these relationships. The non-monophyly of Scelimeninae was strongly supported in all trees, which confirmed the findings of Lin et al. (2015) based on the COI gene with lower node support. Within the traditional subfamily Scelimeninae, the eight species sampled in this study represent eight genera (three main tribes), and the topologies were almost consistent across two datasets, except for the position of Scelimenini (Figs. 5 and 6, yellow box). The PCG123 analysis showed that the three species of Scelimenini were at the base of the clade consisting of Tetriginae, *S. spicupennis* (Metrodorinae) and *T. bufo* (Cladonotinae). The topologies, based on two datasets, placed *L. prominenoculus* closer to *Y. bannaensis* (Cladonotinae) than two other species from Thoradontini (Figs. 5 and 6, red box). Meanwhile, *C. japonicus* of Criotettigini (Figs. 5 and 6, purple box) was well supported as a sister clade to *B. lativertex* (Metrodorinae) by all the results. In addition, *Z. curvispinus* was recognized as the earliest diverging species among the analyzed tetrigid species in all analyses. In the new Orthoptera Species File (OSF), two tribes (Criotettigini and Thoradontini) were excluded from Scelimeninae (Adžić et al., 2020; Cigliano et al., 2020). Only the tribe Scelimenini and three genera of *Dengonius*, *Hebarditettix*, and *Zhengitettix* are still regarded as Scelimeninae members in the modern OSF taxonomy. Similarly, four species (three within Scelimenini and one within *Zhengitettix*) of re-defined Scelimeninae were still clustered as non-monophyletic groups in all analyses (Figs. 5 and 6; yellow and green box).

All phylogenetic analyses revealed that Tetriginae was a monophyletic group and was more recently derived than other analyzed groups, which supported the results of previous phylogenetic studies on the basis of one or several genes (Jiang, 2000; Lin et al., 2015). For traditional Scelimentinae, our phylogenetic trees showed that three species of Scelimenini clustered together, three species of Thoradontini and *Y. bannaensis* (Cladonotinae) formed a group, the species from Criotettigini and *B. lativertex* (Metrodorinae) were the sister group, and *Z. curvispinus* was at the base of the clade consisting of all other tetrigid species. The above relationships supported the opinions of Adžić et al. (2020) that spiky pygmy grasshoppers (Scelimeninae) are not monophyletic, but more likely polyphyletic. While our results confirmed non-monophyly of Scelimeninae based on both (old and new) OSF taxonomy, greater mitogenomic taxon sampling is needed to resolve many questions concerning the phylogenetic relationships among Scelimeninae, and Tetrigidae as a whole.

## Conclusion

We sequenced eight nearly complete mitochondrial genome sequences of Scelimeninae. The newly sequenced mitogenomes shared similar gene arrangement: the order of tRNA genes between COII and ATP8 was reversed compared with the ancestral order of insects. Notably, the longest intergenic spacer (IGS) region in the mitogenomes was found between tRNA^Ser(UCN)^ and ND1. Our phylogenetic analyses all supported the non-monophyletic Scelimeninae, whereas Tetriginae was reconfirmed as a monophyletic group based on current mitogenome data. A lack of solid molecular information has restricted the understanding of the evolution and phylogeny of Tetrigidae. In this context, the addition of taxa and newly sequenced mitogenomes will contribute to the further study of evolution and phylogeny of among Tetrigidae.

## Supplemental Information

10.7717/peerj.10523/supp-1 Supplemental Information 1 The species used for phylogenetic trees in this study.

10.7717/peerj.10523/supp-2 Supplemental Information 2 Annotation and gene organization of the new mitogenomes.

10.7717/peerj.10523/supp-3 Supplemental Information 3 Start and stop codons of PCGs in the mitogenomes of *C. japonicus* (CJ), *F. longicornis* (FL), *Z. curvispinus* (ZC), *L. prominenoculus* (LP), *E. oculatus* (EO), *T. nodulosa* (TN), *S. melli* (SM), *P. sichuanense* (PS).

10.7717/peerj.10523/supp-4 Supplemental Information 4 Relative synonymous codon usage (RSCU) of new mitogenomes.

10.7717/peerj.10523/supp-5 Supplemental Information 5 The sequences of eight new mitogenomes.

## Additional Information and Declarations

### DNA Deposition

The following information was supplied regarding the deposition of DNA sequences: The sequences of eight new mitogenomes are available at NCBI: MT162542–MT162549.

### Data Availability

The following information was supplied regarding data availability: The sequences are available in the Supplemental Files.
