# Supplementary material for: Mitochondrial genomes of eight Scelimeninae species (Orthoptera) and their phylogenetic implications within Tetrigoidea
Source: PeerJ. 2021 Feb 2;9:e10523. doi: 10.7717/peerj.10523 (PMC7863789; doi:10.7717/peerj.10523)
Supplement: Supplemental Information 1 [file peerj-09-10523-s001.docx]

| **Superfamily** | **Family** | **Subfamily** | **Species** | **Genbank No.** |
| --- | --- | --- | --- | --- |
| INGROUP |  |  |  |  |
| Tetrigoidea | Tetrigidae | Cladonotinae | *Trachytettix bufo* | JX913766 |
|  |  |  | *Yunnantettix bannaensis* | MN083181 |
|  |  | Metrodorinae | *Bolivaritettix lativertex* | MN083173 |
|  |  |  | *Systolederus spicupennis* | MH791445 |
|  |  | Tetriginae | *Alulatettix yunnanensis* | JQ272702 |
|  |  |  | *Euparatettix variabilis* | MN083178 |
|  |  |  | *Euparatettix bimaculatus* | MN083177 |
|  |  |  | *Ergatettix dorsifera* | MN083175 |
|  |  |  | *Coptotettix longjiangensis* | KY798413 |
|  |  |  | *Formosatettix qinlingensis* | KY798412 |
|  |  |  | *Tetrix ruyuanensis* | MN083180 |
|  |  |  | *Tetrix japonica* | JQ340002 |
|  |  | Scelimeninae | *Criotettix japonicus* | This study |
|  |  |  | *Falconius longicornis* | This study |
|  |  |  | *Zhengitettix curvispinus* | This study |
|  |  |  | *Loxilobus prominenoculus* | This study |
|  |  |  | *Eucriotettix oculatus* | This study |
|  |  |  | *Thoradonta nodulosa* | This study |
|  |  |  | *Scelimena melli* | This study |
|  |  |  | *Paragavialidium sichuanense* | This study |
| OUTGROUP |  |  |  |  |
| Tridactyloidea | Ripipterygidae | Ripipteryginae | *Mirhipipteryx andensis* | KM657340 |
|  | Tridactylidae | Tridactylinae | *Ellipes miuntus* | GU945502 |

**Table S1** The species used for phylogenetic trees in this study.
