# Supplementary material for: Mitochondrial genomes of eight Scelimeninae species (Orthoptera) and their phylogenetic implications within Tetrigoidea
Source: PeerJ. 2021 Feb 2;9:e10523. doi: 10.7717/peerj.10523 (PMC7863789; doi:10.7717/peerj.10523)
Supplement: Supplemental Information 2 [file peerj-09-10523-s002.docx]

**Table S2** Annotation and gene organization of the *C. japonicus* mitogenome.

| Gene | Strand | Nucleotide no. | Size (bp) | IN | Anticodon | Start codon | Stop codon |
| --- | --- | --- | --- | --- | --- | --- | --- |
| - | J | <1-5 | - | - | - | - | - |
| ND2  tRNATrp  tRNACys | J  J  N | 6-1007  1006-1063  1065-1126 | 1002  58  62 | -  -2  1 | -  TCA  GCA | ATT  - | TAA  - |
| tRNATyr | N | 1127-1189 | 63 | 0 | GTA | - | - |
| COI | J | 1187-2725 | 1539 | -3 | - | ATC | TAA |
| tRNALeu(UUR) | J | 2721-2783 | 63 | -5 | TAA | - | - |
| COII | J | 2785-3462 | 678 | 1 | - | ATG | TAA |
| tRNAAsp | J | 3461-3529 | 69 | -2 | GTC | - | - |
| tRNALys | J | 3531-3598 | 68 | 1 | CTT | - | - |
| ATP8 | J | 3599-3754 | 156 | 0 | - | ATG | TAA |
| ATP6 | J | 3748-4419 | 672 | -7 | - | ATG | TAA |
| COIII | J | 4419-5207 | 789 | -1 | - | ATG | TAA |
| tRNAGly | J | 5207-5268 | 62 | 0 | TCC | - | - |
| ND3 | J | 5266-5622 | 357 | -3 | - | GTA | TAA |
| tRNAAla | J | 5622-5685 | 64 | -1 | TGC | - | - |
| tRNAArg | J | 5686-5746 | 61 | 0 | TCG | - | - |
| tRNAAsn | J | 5747-5809 | 63 | 0 | GTT | - | - |
| tRNASer(AGN) | J | 5810-5876 | 67 | 0 | GCT | - | - |
| tRNAGlu | J | 5877-5940 | 64 | 0 | TTC | - | - |
| tRNAPhe | N | 5939-6001 | 63 | -2 | GAA | - | - |
| ND5 | N | 6001-7715 | 1715 | -1 | - | ATG | TA |
| tRNAHis | N | 7717-7779 | 63 | 1 | GTG | - | - |
| ND4 | N | 7779-9098 | 1320 | -1 | - | ATG | TAG |
| ND4L | N | 9092-9376 | 285 | -7 | - | ATT | TAA |
| tRNAThr | J | 9379-9443 | 65 | 2 | TGT | - | - |
| tRNAPro | N | 9444-9508 | 65 | 0 | TGG | - | - |
| ND6 | J | 9510-10001 | 492 | 1 | - | TTG | TAA |
| Cytb | J | 10001-11134 | 1134 | -1 | - | ATG | TAA |
| tRNASer(UCN) | J | 11133-11199 | 67 | -2 | TGA | - | - |
| ND1 | N | 11290-12240 | 951 | 90 | - | ATA | TAA |
| tRNALeu(CUN) | N | 12235-12297 | 63 | -6 | TAG | - | - |
| 16S | N | 12299-13595 | 1297 | 1 | - | - | - |
| tRNAVal | N | 13598-13666 | 69 | 2 | TAC | - | - |
| 12S | N | 13668->14247 | >580 | 1 | - | - | - |

**Table S3** Annotation and gene organization of the *F. longicornis* mitogenome.

| Gene | Strand | Nucleotide no. | Size (bp) | IN | Anticodon | Start codon | Stop codon |
| --- | --- | --- | --- | --- | --- | --- | --- |
| tRNA^Gln^ | N | <1-58 | >58 | 0 | TTG | - | - |
| tRNA^Met^ | J | 58-125 | 68 | -1 | CAT | - | - |
| ND2  tRNA^Trp^  tRNA^Cys^ | J  J  N | 126-1133  1132-1197  1190-1251 | 1008  66  62 | 0  -2  -8 | -  TCA  GCA | ATA  - | TAA  - |
| COI | J | 1273-2811 | 1539 | 21 | - | ATC | TAA |
| tRNA^Leu(UUR)^ | J | 2807-2870 | 64 | -5 | TAA | - | - |
| COII | J | 2873-3556 | 684 | 2 | - | ATG | TAA |
| tRNA^Asp^ | J | 3555-3616 | 62 | -2 | GTC | - | - |
| tRNA^Lys^ | J | 3618-3687 | 70 | 1 | CTT | - | - |
| ATP8 | J | 3691-3849 | 159 | 3 | - | ATG | TAA |
| ATP6 | J | 3843-4514 | 672 | -7 | - | ATG | TAA |
| COIII | J | 4514-5302 | 789 | -1 | - | ATG | TAA |
| tRNA^Gly^ | J | 5303-5366 | 64 | 0 | TCC | - | - |
| ND3 | J | 5367-5720 | 354 | 0 | - | ATA | TAG |
| tRNA^Ala^ | J | 5719-5782 | 64 | -2 | TGC | - | - |
| tRNA^Arg^ | J | 5783-5849 | 67 | 0 | TCG | - | - |
| tRNA^Asn^ | J | 5850-5913 | 64 | 0 | GTT | - | - |
| tRNA^Ser(AGN)^ | J | 5914-5980 | 67 | 0 | GCT | - | - |
| tRNA^Glu^ | J | 5981-6044 | 64 | 0 | TTC | - | - |
| tRNA^Phe^ | N | 6043-6107 | 65 | -2 | GAA | - | - |
| ND5 | N | 6111-7830 | 1720 | 3 | - | ATG | T |
| tRNA^His^ | N | 7834-7897 | 64 | 3 | GTG | - | - |
| ND4 | N | 7897-9222 | 1326 | -1 | - | ATG | TAG |
| ND4L | N | 9216-9506 | 291 | -7 | - | ATT | TAA |
| tRNA^Thr^ | J | 9509-9574 | 66 | 2 | TGT | - | - |
| tRNA^Pro^ | N | 9575-9637 | 63 | 0 | TGG | - | - |
| ND6 | J | 9639-10139 | 501 | 1 | - | ATG | TAA |
| Cytb | J | 10139-11278 | 1140 | -1 | - | ATG | TAG |
| tRNA^Ser(UCN)^ | J | 11277-11342 | 66 | -2 | TGA | - | - |
| ND1 | N | 11985-12923 | 939 | 642 | - | ATT | TAA |
| tRNA^Leu(CUN)^ | N | 12924-12989 | 66 | 0 | TAG | - | - |
| 16S | N | 12990-14280 | 1291 | 0 | - | - | - |
| tRNA^Val^ | N | 14282-14353 | 72 | 1 | TAC | - | - |
| 12S  A+T-rich | N  J | 14354-15131  15132->15566 | 778  >435 | 0  0 | -  - | -  - | -  - |

**Table S4** Annotation and gene organization of the *Z. curvispinus* mitogenome.

| **Gene** | **Strand** | **Nucleotide no.** | **Size (bp)** | **IN** | **Anticodon** | **Start codon** | **Stop codon** |
| --- | --- | --- | --- | --- | --- | --- | --- |
| tRNA^Met^ | J | <1-40 | >40 | - | CAT | - | - |
| ND2  tRNA^Trp^  tRNA^Cys^ | J  J  N | 41-1045  1044-1107  1100-1162 | 1005  64  63 | -  -2  2 | -  TCA  GCA | ATT  - | TAA  - |
| tRNA^Tyr^ | N | 1163-1225 | 63 | 0 | GTA | - | - |
| COI | J | 1223-2788 | 1566 | -3 | - | ATC | TAA |
| tRNA^Leu(UUR)^ | J | 2757-2820 | 64 | -32 | TAA | - | - |
| COII | J | 2822-3502 | 681 | 1 | - | ATG | TAA |
| tRNA^Asp^ | J | 3501-3562 | 62 | -2 | GTC | - | - |
| tRNA^Lys^ | J | 3564-3629 | 66 | 1 | CTT | - | - |
| ATP8 | J | 3633-3791 | 159 | 3 | - | ATG | TAA |
| ATP6 | J | 3785-4453 | 669 | -7 | - | ATG | TAA |
| COIII | J | 4453-5242 | 790 | -1 | - | ATG | T |
| tRNA^Gly^ | J | 5243-5305 | 63 | 0 | TCC | - | - |
| ND3 | J | 5303-5659 | 357 | -3 | - | ATA | TAG |
| tRNA^Ala^ | J | 5658-5722 | 65 | -2 | TGC | - | - |
| tRNA^Arg^ | J | 5723-5786 | 64 | 0 | TCG | - | - |
| tRNA^Asn^ | J | 5787-5853 | 67 | 0 | GTT | - | - |
| tRNA^Ser(AGN)^ | J | 5854-5921 | 68 | 0 | GCT | - | - |
| tRNA^Glu^ | J | 5922-5985 | 64 | 0 | TTC | - | - |
| tRNA^Phe^ | N | 5984-6045 | 62 | -2 | GAA | - | - |
| ND5 | N | 6052-7771 | 1720 | 6 | - | ATG | TAA |
| tRNA^His^ | N | 7773-7837 | 65 | 1 | GTG | - | - |
| ND4 | N | 7837-9156 | 1320 | -1 | - | ATG | TAA |
| ND4L | N | 9150-9440 | 291 | -7 | - | ATT | TAA |
| tRNA^Thr^ | J | 9443-9506 | 64 | 2 | TGT | - | - |
| tRNA^Pro^ | N | 9507-9568 | 62 | 0 | TGG | - | - |
| ND6 | J | 9571-10071 | 501 | 2 | - | ATC | TAA |
| Cytb | J | 10075-11214 | 1140 | 3 | - | ATG | TAA |
| tRNA^Ser(UCN)^ | J | 11213-11277 | 65 | -2 | TGA | - | - |
| ND1 | N | 12223-13173 | 951 | 945 | - | ATT | TAA |
| tRNA^Leu(CUN)^ | N | 13174-13236 | 63 | 0 | TAG | - | - |
| 16S | N | 13238-14530 | 1293 | 1 | - | - | - |
| tRNA^Val^ | N | 14529-14601 | 73 | -2 | TAC | - | - |
| 12S  A+T-rich | N  J | 14602-15382  15383->16380 | 781  >1278 | 0  0 | -  - | -  - | -  - |

**Table S5** Annotation and gene organization of the *L. prominenoculus* mitogenome.

| **Gene** | **Strand** | **Nucleotide no.** | **Size (bp)** | **IN** | **Anticodon** | **Start codon** | **Stop codon** |
| --- | --- | --- | --- | --- | --- | --- | --- |
| - | - | <1-8 | - | - | - | - | - |
| ND2  tRNA^Trp^  tRNA^Cys^ | J  J  N | 9-1019  1018-1084  1077-1138 | 1011  67  62 | -  -2  -8 | -  TCA  GCA | ATC  - | TAA  - |
| tRNA^Tyr^ | N | 1139-1201 | 63 | 0 | GTA | - | - |
| COI | J | 1199-2737 | 1539 | -3 | - | ATC | TAA |
| tRNA^Leu(UUR)^ | J | 2733-2795 | 63 | -5 | TAA | - | - |
| COII | J | 2796-3470 | 675 | 0 | - | ATG | TAA |
| tRNA^Asp^ | J | 3469-3532 | 64 | -2 | GTC | - | - |
| tRNA^Lys^ | J | 3534-3598 | 65 | 1 | CTT | - | - |
| ATP8 | J | 3600-3755 | 156 | 1 | - | ATG | TAA |
| ATP6 | J | 3749-4420 | 672 | -7 | - | ATG | TAA |
| COIII | J | 4420-5211 | 792 | -1 | - | ATG | TAA |
| tRNA^Gly^ | J | 5211-5272 | 62 | -1 | TCC | - | - |
| ND3 | J | 5273-5626 | 354 | 0 | - | ATC | TAG |
| tRNA^Ala^ | J | 5625-5688 | 64 | -2 | TGC | - | - |
| tRNA^Arg^ | J | 5689-5749 | 61 | 0 | TCG | - | - |
| tRNA^Asn^ | J | 5750-5813 | 64 | 0 | GTT | - | - |
| tRNA^Ser(AGN)^ | J | 5814-5880 | 67 | 0 | GCT | - | - |
| tRNA^Glu^ | J | 5881-5942 | 62 | 0 | TTC | - | - |
| tRNA^Phe^ | N | 5941-6002 | 62 | -2 | GAA | - | - |
| ND5 | N | 6009-7724 | 1716 | 6 | - | ATG | TAA |
| tRNA^His^ | N | 7726-7788 | 63 | 1 | GTG | - | - |
| ND4 | N | 7788-9113 | 1326 | -1 | - | ATG | TAG |
| ND4L | N | 9107-9391 | 285 | -7 | - | ATT | TAA |
| tRNA^Thr^ | J | 9394-9455 | 62 | 2 | TGT | - | - |
| tRNA^Pro^ | N | 9456-9520 | 65 | 0 | TGG | - | - |
| ND6 | J | 9522-10016 | 495 | 1 | - | TTG | TAA |
| Cytb | J | 10016-11152 | 1137 | -1 | - | ATG | TAA |
| tRNA^Ser(UCN)^ | J | 11151-11216 | 66 | -2 | TGA | - | - |
| ND1 | N | 12079-13032 | 954 | 862 | - | ATA | TAA |
| tRNA^Leu(CUN)^ | N | 13021-13084 | 64 | -12 | TAG | - | - |
| 16S | N | 13086-14371 | 1286 | 1 | - | - | - |
| tRNA^Val^ | N | 14372-14438 | 67 | 0 | TAC | - | - |
| 12S | NJ | 14441->15025 | >585 | 2 | - | - | - |

**Table S6** Annotation and gene organization of the *E. oculatus* mitogenome.

| **Gene** | **Strand** | **Nucleotide no.** | **Size (bp)** | **IN** | **Anticodon** | **Start codon** | **Stop codon** |
| --- | --- | --- | --- | --- | --- | --- | --- |
| - | - | <1-3 | - | - | - | - | - |
| ND2  tRNA^Trp^  tRNA^Cys^ | J  J  N | 4-1014  1013-1075  1068-1130 | 1011  63  63 | -  -2  -8 | -  TCA  GCA | ATC  - | TAA  - |
| tRNA^Tyr^ | N | 1131-1193 | 63 | 0 | GTA | - | - |
| COI | J | 1191-2729 | 1539 | -3 | - | ATC | TAA |
| tRNA^Leu(UUR)^ | J | 2725-2787 | 63 | -5 | TAA | - | - |
| COII | J | 2788-3465 | 678 | 0 | - | ATG | TAA |
| tRNA^Asp^ | J | 3464-3526 | 63 | -2 | GTC | - | - |
| tRNA^Lys^ | J | 3528-3593 | 66 | 1 | CTT | - | - |
| ATP8 | J | 3594-3749 | 156 | 0 | - | ATA | TAA |
| ATP6 | J | 3743-4414 | 672 | -7 | - | ATG | TAA |
| COIII | J | 4414-5205 | 792 | -1 | - | ATG | TAG |
| tRNA^Gly^ | J | 5205-5267 | 63 | -1 | TCC | - | - |
| ND3 | J | 5268-5621 | 354 | 0 | - | ATT | TAG |
| tRNA^Ala^ | J | 5620-5683 | 64 | -2 | TGC | - | - |
| tRNA^Arg^ | J | 5684-5744 | 61 | 0 | TCG | - | - |
| tRNA^Asn^ | J | 5745-5808 | 64 | 0 | GTT | - | - |
| tRNA^Ser(AGN)^ | J | 5809-5875 | 67 | 0 | GCT | - | - |
| tRNA^Glu^ | J | 5876-5938 | 63 | 0 | TTC | - | - |
| tRNA^Phe^ | N | 5937-5999 | 63 | -2 | GAA | - | - |
| ND5 | N | 5995-7716 | 1722 | -5 | - | TTG | TAA |
| tRNA^His^ | N | 7718-7782 | 65 | 1 | GTG | - | - |
| ND4 | N | 7782-9107 | 1326 | -1 | - | ATG | TAA |
| ND4L | N | 9101-9385 | 285 | -7 | - | ATT | TAA |
| tRNA^Thr^ | J | 9388-9449 | 62 | 2 | TGT | - | - |
| tRNA^Pro^ | N | 9450-9514 | 65 | 0 | TGG | - | - |
| ND6 | J | 9516-10007 | 492 | 1 | - | TTG | TAA |
| Cytb | J | 10007-11143 | 1137 | -1 | - | ATG | TAA |
| tRNA^Ser(UCN)^ | J | 11142-11206 | 65 | -2 | TGA | - | - |
| ND1 | N | 11916-12866 | 951 | 709 | - | ATA | TAA |
| tRNA^Leu(CUN)^ | N | 12855-12921 | 67 | -12 | TAG | - | - |
| 16S | N | 12923-14199 | 1277 | 1 | - | - | - |
| tRNA^Val^ | N | 14207-14274 | 68 | 17 | TAC | - | - |
| 12S | N | 14276->14838 | >563 | 1 | - | - | - |

**Table S7** Annotation and gene organization of the *T. nodulosa* mitogenome.

| **Gene** | **Strand** | **Nucleotide no.** | **Size (bp)** | **IN** | **Anticodon** | **Start codon** | **Stop codon** |
| --- | --- | --- | --- | --- | --- | --- | --- |
| tRNA^Gln^ | N | <1-60 | >60 | - | TTG | - | - |
| tRNA^Met^ | J | 61-128 | 68 | 0 | CAT | - | - |
| ND2  tRNA^Trp^  tRNA^Cys^ | J  J  N | 129-1139  1138-1203  1196-1257 | 1011  66  62 | 0  -2  -8 | -  TCA  GCA | ATT  - | TAA  - |
| tRNA^Tyr^ | N | 1258-1324 | 67 | 0 | GTA | - | - |
| COI | J | 1322-2860 | 1539 | -3 | - | ATC | TAG |
| tRNA^Leu(UUR)^ | J | 2856-2918 | 63 | -5 | TAA | - | - |
| COII | J | 2919-3596 | 678 | 0 | - | ATG | TAA |
| tRNA^Asp^ | J | 3595-3657 | 63 | -2 | GTC | - | - |
| tRNA^Lys^ | J | 3658-3723 | 66 | 0 | CTT | - | - |
| ATP8 | J | 3724-3876 | 153 | 0 | - | ATG | TAA |
| ATP6 | J | 3870-4541 | 672 | -7 | - | ATG | TAA |
| COIII | J | 4541-5332 | 792 | -1 | - | ATG | TAA |
| tRNA^Gly^ | J | 5332-5393 | 62 | -1 | TCC | - | - |
| ND3 | J | 5391-5747 | 357 | -3 | - | ATA | TAG |
| tRNA^Ala^ | J | 5746-5809 | 64 | -2 | TGC | - | - |
| tRNA^Arg^ | J | 5810-5868 | 59 | 0 | TCG | - | - |
| tRNA^Asn^ | J | 5865-5928 | 64 | -4 | GTT | - | - |
| tRNA^Ser(AGN)^ | J | 5929-5995 | 67 | 0 | GCT | - | - |
| tRNA^Glu^ | J | 5996-6058 | 63 | 0 | TTC | - | - |
| tRNA^Phe^ | N | 6057-6118 | 62 | -2 | GAA | - | - |
| ND5 | N | 6114-7835 | 1722 | -5 | - | ATG | TAA |
| tRNA^His^ | N | 7837-7898 | 62 | 1 | GTG | - | - |
| ND4 | N | 7898-9223 | 1326 | -1 | - | ATG | TAG |
| ND4L | N | 9217-9501 | 285 | -7 | - | ATT | TAA |
| tRNA^Thr^ | J | 9504-9565 | 62 | 2 | TGT | - | - |
| tRNA^Pro^ | N | 9566-9630 | 65 | 0 | TGG | - | - |
| ND6 | J | 9632-10126 | 495 | 1 | - | TTG | TAA |
| Cytb | J | 10126-11262 | 1137 | -1 | - | ATG | TAA |
| tRNA^Ser(UCN)^ | J | 11261-11327 | 67 | -2 | TGA | - | - |
| ND1 | N | 11727-12671 | 945 | 399 | - | ATT | TAA |
| tRNA^Leu(CUN)^ | N | 12672-12736 | 65 | 0 | TAG | - | - |
| 16S | N | 12738-14020 | 1283 | 1 | - | - | - |
| tRNA^Val^ | N | 14026-14092 | 67 | 5 | TAC | - | - |
| 12S | N | 14095->14759 | >665 | 2 | - | - | - |

**Table S8** Annotation and gene organization of the *S. melli* Gunther mitogenome.

| **Gene** | **Strand** | **Nucleotide no.** | **Size (bp)** | **IN** | **Anticodon** | **Start codon** | **Stop codon** |
| --- | --- | --- | --- | --- | --- | --- | --- |
| tRNA^Gln^ | N | <1-59 | >59 | - | TTG | - | - |
| tRNA^Met^ | J | 59-127 | 69 | -1 | CAT | - | - |
| ND2  tRNA^Trp^  tRNA^Cys^ | J  J  N | 128-1141  1140-1205  1198-1260 | 1014  66  63 | 0  -2  -8 | -  TCA  GCA | ATC  - | TAA  - |
| tRNA^Tyr^ | N | 1261-1327 | 67 | 0 | GTA | - | - |
| COI | J | 1325-2863 | 1539 | -3 | - | ATC | TAA |
| tRNA^Leu(UUR)^ | J | 2859-2922 | 64 | -5 | TAA | - | - |
| COII | J | 2923-3603 | 681 | 0 | - | ATG | TAA |
| tRNA^Asp^ | J | 3604-3665 | 62 | 0 | GTC | - | - |
| tRNA^Lys^ | J | 3667-3736 | 70 | 1 | CTT | - | - |
| ATP8 | J | 3739-3897 | 159 | 2 | - | ATG | TAA |
| ATP6 | J | 3891-4562 | 672 | -7 | - | ATG | TAA |
| COIII | J | 4562-5350 | 789 | -1 | - | ATG | T |
| tRNA^Gly^ | J | 5350-5412 | 63 | -1 | TCC | - | - |
| ND3 | J | 5413-5766 | 354 | 0 | - | ATT | TAG |
| tRNA^Ala^ | J | 5765-5827 | 63 | -2 | TGC | - | - |
| tRNA^Arg^ | J | 5900-5961 | 62 | 72 | TCG | - | - |
| tRNA^Asn^ | J | 5962-6027 | 66 | 0 | GTT | - | - |
| tRNA^Ser(AGN)^ | J | 6028-6094 | 67 | 0 | GCT | - | - |
| tRNA^Glu^ | J | 6095-6157 | 63 | 0 | TTC | - | - |
| tRNA^Phe^ | N | 6156-6217 | 62 | -2 | GAA | - | - |
| ND5 | N | 6218-7943 | 1726 | 0 | - | ATG | TAA |
| tRNA^His^ | N | 7945-8009 | 65 | 1 | GTG | - | - |
| ND4 | N | 8009-9334 | 1326 | -1 | - | ATG | TAA |
| ND4L | N | 9328-9615 | 288 | -7 | - | ATT | TAA |
| tRNA^Thr^ | J | 9618-9682 | 65 | 2 | TGT | - | - |
| tRNA^Pro^ | N | 9683-9746 | 64 | 0 | TGG | - | - |
| ND6 | J | 9748-10248 | 501 | 1 | - | TTG | TAA |
| Cytb | J | 10248-11387 | 1140 | -1 | - | ATG | TAG |
| tRNA^Ser(UCN)^ | J | 11386-11451 | 66 | -2 | TGA | - | - |
| ND1 | N | 11465-12412 | 948 | 15 | - | ATA | TAA |
| tRNA^Leu(CUN)^ | N | 12407-12471 | 65 | -6 | TAG | - | - |
| 16S | N | 12473-13767 | 1295 | 1 | - | - | - |
| tRNA^Val^ | N | 13768-13834 | 67 | 0 | TAC | - | - |
| 12S | N | 13835->14408 | >574 | 0 | - | - | - |

**Table S9** Annotation and gene organization of the *P. sichuanensis* mitogenome.

| **Gene** | **Strand** | **Nucleotide no.** | **Size (bp)** | **IN** | **Anticodon** | **Start codon** | **Stop codon** |
| --- | --- | --- | --- | --- | --- | --- | --- |
| - | J | <1-4 | - | - | - | - | - |
| ND2  tRNA^Trp^  tRNA^Cys^ | J  J  N | 5-1015  1014-1077  1070-1131 | 1011  64  62 | -  -2  -8 | -  TCA  GCA | ATT  - | TAA  - |
| tRNA^Tyr^ | N | 1132-1196 | 65 | 0 | GTA | - | - |
| COI | J | 1194-2732 | 1539 | -3 | - | ATC | TAA |
| tRNA^Leu(UUR)^ | J | 2728-2792 | 65 | -5 | TAA | - | - |
| COII | J | 2794-3474 | 681 | 1 | - | ATG | TAA |
| tRNA^Asp^ | J | 3475-3537 | 63 | 0 | GTC | - | - |
| tRNA^Lys^ | J | 3539-3607 | 69 | 1 | CTT | - | - |
| ATP8 | J | 3612-3770 | 159 | 4 | - | ATG | TAA |
| ATP6 | J | 3764-4432 | 669 | -7 | - | ATG | TAA |
| COIII | J | 4432-5215 | 784 | -1 | - | ATG | T |
| tRNA^Gly^ | J | 5216-5279 | 64 | 0 | TCC | - | - |
| ND3 | J | 5274-5633 | 360 | -6 | - | ATT | TAG |
| tRNA^Ala^ | J | 5632-5695 | 64 | -2 | TGC | - | - |
| tRNA^Arg^ | J | 5696-5759 | 64 | 0 | TCG | - | - |
| tRNA^Asn^ | J | 5761-5824 | 64 | 1 | GTT | - | - |
| tRNA^Ser(AGN)^ | J | 5825-5891 | 67 | 0 | GCT | - | - |
| tRNA^Glu^ | J | 5892-5955 | 64 | 0 | TTC | - | - |
| tRNA^Phe^ | N | 5954-6014 | 61 | -2 | GAA | - | - |
| ND5 | N | 6015-7740 | 1726 | 0 | - | TTG | T |
| tRNA^His^ | N | 7741-7805 | 65 | 0 | GTG | - | - |
| ND4 | N | 7805-9130 | 1326 | -1 | - | ATG | TAG |
| ND4L | N | 9124-9414 | 291 | -7 | - | ATT | TAA |
| tRNA^Thr^ | J | 9417-9479 | 63 | 2 | TGT | - | - |
| tRNA^Pro^ | N | 9480-9544 | 65 | 0 | TGG | - | - |
| ND6 | J | 9546-10043 | 498 | 1 | - | ATG | TAA |
| Cytb | J | 10043-11182 | 1140 | -1 | - | ATG | TAG |
| tRNA^Ser(UCN)^ | J | 11181-11246 | 66 | -2 | TGA | - | - |
| ND1 | N | 11258-12199 | 942 | 11 | - | ATT | TAA |
| tRNA^Leu(CUN)^ | N | 12200-12264 | 65 | 0 | TAG | - | - |
| 16S | N | 12266->13112 | >850 | 1 | - | - | - |
