# Supplementary material for: Mitochondrial genomes of eight Scelimeninae species (Orthoptera) and their phylogenetic implications within Tetrigoidea
Source: PeerJ. 2021 Feb 2;9:e10523. doi: 10.7717/peerj.10523 (PMC7863789; doi:10.7717/peerj.10523)
Supplement: Supplemental Information 3 [file peerj-09-10523-s003.docx]

**Table S10** Start and stop codons of PCGs in the mitogenomes of *C. japonicus* (CJ), *F. longicornis* (FL), *Z. curvispinus* (ZC), *L. prominenoculus* (LP), *E. oculatus* (EO), *T. nodulosa* (TN), *S. melli* (SM), *P. sichuanense* (PS), *T. bufo* (TB), *Y. bannaensis* (YB), *B. lativertex* (BL), *S. spicupennis* (SS), *A. yunnanensis* (AY), *C. longjiangensis* (CL), *F. qinlingensis* (FQ), *E. dorsifera* (ED), *E. bimaculatus* (EB), *E. variabilis* (EV), *T. ruyuanensis* (TR), *T. japonica* (TJ).

| Species | Gene |  |  |  |  |  |  |  |  |  |  |  |  |
| --- | --- | --- | --- | --- | --- | --- | --- | --- | --- | --- | --- | --- | --- |
|  | ATP6 | ATP8 | COI | COII | COIII | CYTB | ND1 | ND2 | ND3 | ND4 | ND4L | ND5 | ND6 |
| CJ | ATG/TAA | ATG/TAA | ATC/TAA | ATG/TAA | ATG/TAA | ATG/TAA | ATA/TAA | ATT/TAA | GTA/TAA | ATG/TAG | ATT/TAA | ATG/T | TTG/TAA |
| FL | ATG/TAA | ATG/TAA | ATC/TAA | ATG/TAA | ATG/TAA | ATG/TAG | ATT/TAA | ATA/TAA | ATA/TAG | ATG/TAG | ATT/TAA | ATG/T | ATG/TAA |
| ZC | ATG/TAA | ATG/TAA | ATC/TAA | ATG/TAA | ATG/T | ATG/TAA | ATT/TAA | ATT/TAA | ATA/TAG | ATG/TAA | ATT/TAA | ATG/T | ATC/TAA |
| LP | ATG/TAA | ATG/TAA | ATC/TAA | ATG/TAA | ATG/TAA | ATG/TAA | ATA/TAA | ATC/TAA | ATC/TAG | ATG/TAG | ATT/TAA | ATG/TAA | TTG/TAA |
| EO | ATG/TAA | ATA/TAA | ATC/TAA | ATG/TAA | ATG/TAG | ATG/TAA | ATA/TAA | ATC/TAA | ATT/TAG | ATG/TAA | ATT/TAA | ATG/TAA | TTG/TAA |
| TN | ATG/TAA | ATG/TAA | ATC/TAG | ATG/TAA | ATG/TAA | ATG/TAA | ATT/TAA | ATT/TAA | ATA/TAG | ATG/TAG | ATT/TAA | ATG/TAA | TTG/TAA |
| SM | ATG/TAA | ATG/TAA | ATC/TAA | ATG/TAA | ATG/T | ATG/TAG | ATA/TAA | ATC/TAA | ATT/TAG | ATG/TAA | ATT/TAA | ATG/T | TTG/TAA |
| PS | ATG/TAA | ATG/TAA | ATC/TAA | ATG/TAA | ATG/T | ATG/TAG | ATT/TAA | ATT/TAA | ATT/TAG | ATG/TAG | ATT/TAA | TTG/T | ATG/TAA |
| TB | ATG/TAA | ATG/TAA | ATC/T | GTG/TAA | ATG/T | ATG/TAG | ATT/TAG | ATG/TAA | ATT/TAG | ATG/TAG | ATT/TAA | ATG/T | TTG/TAA |
| YB | ATG/TAA | ATG/TAA | ATC/T | ATG/TAA | ATG/TAA | ATG/TAG | ATT/TAA | ATT/TAA | ATT/TAG | ATG/TAG | ATT/TAA | ATG/T | TTG/TAA |
| BL | ATG/TAA | ATG/TAA | ATC/T | ATG/TAA | ATG/TAG | ATG/TAA | ATT/TAA | ATT/TAA | ATT/TAG | ATG/TAG | ATT/TAA | ATG/T | TTG/TAA |
| SS | ATG/TAA | ATG/TAA | ATC/T | ATG/TAA | ATG/T | ATG/T | ATT/TAA | ATG/TAA | ATT/TAG | ATG/TAG | ATT/TAA | ATG/T | ATT/TAA |
| AY | ATG/TAA | ATG/TAA | ATC/TAA | ATG/TAA | ATG/TAA | ATG/T | ATA/TAA | ATG/T | ATA/TAG | ATG/T | ATT/TAA | ATG/T | ATG/TAA |
| CL | ATG/TAA | ATG/TAA | ATC/T | ATG/TAA | ATG/T | ATG/TAA | ATT/TAA | GTG/TAA | ATA/T | ATG/TAG | ATT/TAA | ATG/T | ATG/TAA |
| FQ | ATG/TAA | ATG/TAA | ATC/T | ATG/TAA | ATG/T | ATG/TAG | ATT/TAA | ATG/TAA | ATT/T | ATG/TAG | ATT/TAA | ATG/T | ATG/TAA |
| ED | ATG/TAA | ATG/TAA | ATC/T | ATG/TAA | ATG/T | ATG/TAG | ATT/TAG | ATG/TAA | ATC/TAG | ATG/TAG | ATT/TAA | ATG/T | ATT/TAA |
| EB | ATG/TAA | ATG/TAA | ATC/T | ATG/TAA | ATG/T | ATG/TAG | ATT/TAA | GTG/TAA | ATC/TAG | ATG/TAG | ATT/TAA | ATG/T | ATT/TAA |
| EV | ATG/TAA | ATG/TAA | ATC/T | ATG/TAA | ATG/T | ATG/TAG | ATT/TAA | GTG/TAA | ATT/TAG | ATG/TAG | ATT/TAA | ATG/T | ATG/TAA |
| TR | ATG/TAA | ATG/TAA | ATC/T | ATG/TAA | ATG/T | ATG/TAG | ATT/TAA | ATG/TAA | ATC/TAG | ATG/TAG | ATT/TAA | ATG/T | ATG/TAA |
| TJ | ATG/TAA | ATG/TAA | ATC/TAA | ATG/TAA | ATG/T | ATG/TAG | ATA/TAA | ATG/TAA | ATA/TAG | ATG/TAG | ATT/TAA | ATG/TAA | ATG/TAA |
