# Supplementary material for: Mitochondrial genomes of eight Scelimeninae species (Orthoptera) and their phylogenetic implications within Tetrigoidea
Source: PeerJ. 2021 Feb 2;9:e10523. doi: 10.7717/peerj.10523 (PMC7863789; doi:10.7717/peerj.10523)
Supplement: Supplemental Information 4 [file peerj-09-10523-s004.docx]

**Table S11** Relative synonymous codon usage (RSCU) of *C. japonicus* mitogenome.

| Codon | Count | RSCU | Codon | Count | RSCU | Codon | Count | RSCU | Codon | Count | RSCU |
| --- | --- | --- | --- | --- | --- | --- | --- | --- | --- | --- | --- |
| UUU(F) | 260 | 1.68 | UCU(S) | 107 | 2.55 | UAU(Y) | 174 | 1.73 | UGU(C) | 39 | 1.66 |
| UUC(F) | 50 | 0.32 | UCC(S) | 15 | 0.36 | UAC(Y) | 27 | 0.27 | UGC(C) | 8 | 0.34 |
| UUA(L) | 394 | 4.27 | UCA(S) | 100 | 2.38 | UAA(*) | 0 | 0 | UGA(W) | 79 | 1.76 |
| UUG(L) | 55 | 0.6 | UCG(S) | 6 | 0.14 | UAG(*) | 0 | 0 | UGG(W) | 11 | 0.24 |
| CUU(L) | 37 | 0.4 | CCU(P) | 63 | 1.91 | CAU(H) | 48 | 1.39 | CGU(R) | 19 | 1.49 |
| CUC(L) | 8 | 0.09 | CCC(P) | 9 | 0.27 | CAC(H) | 21 | 0.61 | CGC(R) | 2 | 0.16 |
| CUA(L) | 53 | 0.58 | CCA(P) | 51 | 1.55 | CAA(Q) | 53 | 1.63 | CGA(R) | 25 | 1.96 |
| CUG(L) | 6 | 0.07 | CCG(P) | 9 | 0.27 | CAG(Q) | 12 | 0.37 | CGG(R) | 5 | 0.39 |
| AUU(I) | 343 | 1.79 | ACU(T) | 61 | 1.47 | AAU(N) | 181 | 1.7 | AGU(S) | 36 | 0.86 |
| AUC(I) | 40 | 0.21 | ACC(T) | 20 | 0.48 | AAC(N) | 32 | 0.3 | AGC(S) | 5 | 0.12 |
| AUA(M) | 267 | 1.75 | ACA(T) | 81 | 1.95 | AAA(K) | 93 | 1.74 | AGA(S) | 64 | 1.52 |
| AUG(M) | 39 | 0.25 | ACG(T) | 4 | 0.1 | AAG(K) | 14 | 0.26 | AGG(S) | 3 | 0.07 |
| GUU(V) | 63 | 1.49 | GCU(A) | 65 | 1.94 | GAU(D) | 56 | 1.62 | GGU(G) | 74 | 1.44 |
| GUC(V) | 7 | 0.17 | GCC(A) | 16 | 0.48 | GAC(D) | 13 | 0.38 | GGC(G) | 14 | 0.27 |
| GUA(V) | 82 | 1.94 | GCA(A) | 48 | 1.43 | GAA(E) | 72 | 1.85 | GGA(G) | 69 | 1.35 |
| GUG(V) | 17 | 0.4 | GCG(A) | 5 | 0.15 | GAG(E) | 6 | 0.15 | GGG(G) | 48 | 0.94 |

**Table S12** Relative synonymous codon usage (RSCU) of *F. longicornis* mitogenome.

| Codon | Count | RSCU | Codon | Count | RSCU | Codon | Count | RSCU | Codon | Count | RSCU |
| --- | --- | --- | --- | --- | --- | --- | --- | --- | --- | --- | --- |
| UUU(F) | 232 | 1.44 | UCU(S) | 101 | 2.21 | UAU(Y) | 136 | 1.52 | UGU(C) | 42 | 1.75 |
| UUC(F) | 90 | 0.56 | UCC(S) | 36 | 0.79 | UAC(Y) | 43 | 0.48 | UGC(C) | 6 | 0.25 |
| UUA(L) | 247 | 2.91 | UCA(S) | 86 | 1.88 | UAA(*) | 0 | 0 | UGA(W) | 74 | 1.57 |
| UUG(L) | 108 | 1.27 | UCG(S) | 13 | 0.28 | UAG(*) | 0 | 0 | UGG(W) | 20 | 0.43 |
| CUU(L) | 51 | 0.6 | CCU(P) | 47 | 1.38 | CAU(H) | 41 | 1.05 | CGU(R) | 25 | 1.96 |
| CUC(L) | 15 | 0.18 | CCC(P) | 23 | 0.68 | CAC(H) | 37 | 0.95 | CGC(R) | 5 | 0.39 |
| CUA(L) | 78 | 0.92 | CCA(P) | 60 | 1.76 | CAA(Q) | 62 | 1.65 | CGA(R) | 17 | 1.33 |
| CUG(L) | 10 | 0.12 | CCG(P) | 6 | 0.18 | CAG(Q) | 13 | 0.35 | CGG(R) | 4 | 0.31 |
| AUU(I) | 229 | 1.34 | ACU(T) | 55 | 1.03 | AAU(N) | 87 | 1.12 | AGU(S) | 33 | 0.72 |
| AUC(I) | 112 | 0.66 | ACC(T) | 63 | 1.18 | AAC(N) | 68 | 0.88 | AGC(S) | 9 | 0.2 |
| AUA(M) | 230 | 1.52 | ACA(T) | 86 | 1.62 | AAA(K) | 88 | 1.76 | AGA(S) | 72 | 1.58 |
| AUG(M) | 72 | 0.48 | ACG(T) | 9 | 0.17 | AAG(K) | 12 | 0.24 | AGG(S) | 15 | 0.33 |
| GUU(V) | 83 | 1.64 | GCU(A) | 65 | 1.61 | GAU(D) | 50 | 1.37 | GGU(G) | 73 | 1.36 |
| GUC(V) | 9 | 0.18 | GCC(A) | 35 | 0.87 | GAC(D) | 23 | 0.63 | GGC(G) | 9 | 0.17 |
| GUA(V) | 77 | 1.52 | GCA(A) | 56 | 1.39 | GAA(E) | 53 | 1.45 | GGA(G) | 84 | 1.57 |
| GUG(V) | 34 | 0.67 | GCG(A) | 5 | 0.12 | GAG(E) | 20 | 0.55 | GGG(G) | 48 | 0.9 |

**Table S13** Relative synonymous codon usage (RSCU) of *Z. curvispinus* mitogenome.

| Codon | Count |  | RSCU | Codon | Count | RSCU | Codon | Count | RSCU | Codon | Count | RSCU |
| --- | --- | --- | --- | --- | --- | --- | --- | --- | --- | --- | --- | --- |
| UUU(F) | 254 |  | 1.6 | UCU(S) | 87 | 1.96 | UAU(Y) | 165 | 1.68 | UGU(C) | 37 | 1.64 |
| UUC(F) | 63 |  | 0.4 | UCC(S) | 12 | 0.27 | UAC(Y) | 31 | 0.32 | UGC(C) | 8 | 0.36 |
| UUA(L) | 354 |  | 4.01 | UCA(S) | 123 | 2.77 | UAA(*) | 0 | 0 | UGA(W) | 83 | 1.8 |
| UUG(L) | 52 |  | 0.59 | UCG(S) | 6 | 0.14 | UAG(*) | 0 | 0 | UGG(W) | 9 | 0.2 |
| CUU(L) | 46 |  | 0.52 | CCU(P) | 69 | 2.16 | CAU(H) | 63 | 1.66 | CGU(R) | 21 | 1.75 |
| CUC(L) | 11 |  | 0.12 | CCC(P) | 16 | 0.5 | CAC(H) | 13 | 0.34 | CGC(R) | 8 | 0.67 |
| CUA(L) | 66 |  | 0.75 | CCA(P) | 38 | 1.19 | CAA(Q) | 52 | 1.7 | CGA(R) | 17 | 1.42 |
| CUG(L) | 1 |  | 0.01 | CCG(P) | 5 | 0.16 | CAG(Q) | 9 | 0.3 | CGG(R) | 2 | 0.17 |
| AUU(I) | 350 |  | 1.77 | ACU(T) | 65 | 1.47 | AAU(N) | 157 | 1.59 | AGU(S) | 35 | 0.79 |
| AUC(I) | 45 |  | 0.23 | ACC(T) | 31 | 0.7 | AAC(N) | 40 | 0.41 | AGC(S) | 4 | 0.09 |
| AUA(M) | 301 |  | 1.78 | ACA(T) | 75 | 1.69 | AAA(K) | 95 | 1.62 | AGA(S) | 82 | 1.85 |
| AUG(M) | 38 |  | 0.22 | ACG(T) | 6 | 0.14 | AAG(K) | 22 | 0.38 | AGG(S) | 6 | 0.14 |
| GUU(V) | 69 |  | 1.85 | GCU(A) | 53 | 1.67 | GAU(D) | 54 | 1.54 | GGU(G) | 90 | 1.71 |
| GUC(V) | 8 |  | 0.21 | GCC(A) | 15 | 0.47 | GAC(D) | 16 | 0.46 | GGC(G) | 16 | 0.3 |
| GUA(V) | 62 |  | 1.66 | GCA(A) | 58 | 1.83 | GAA(E) | 62 | 1.65 | GGA(G) | 65 | 1.23 |
| GUG(V) | 10 |  | 0.27 | GCG(A) | 1 | 0.03 | GAG(E) | 13 | 0.35 | GGG(G) | 40 | 0.76 |

**Table S14** Relative synonymous codon usage (RSCU) of *L. prominenoculus* mitogenome.

| Codon | Count | RSCU | Codon | Count | RSCU | Codon | Count | RSCU | Codon | Count | RSCU |
| --- | --- | --- | --- | --- | --- | --- | --- | --- | --- | --- | --- |
| UUU(F) | 249 | 1.52 | UCU(S) | 112 | 2.47 | UAU(Y) | 147 | 1.61 | UGU(C) | 39 | 1.73 |
| UUC(F) | 78 | 0.48 | UCC(S) | 29 | 0.64 | UAC(Y) | 36 | 0.39 | UGC(C) | 6 | 0.27 |
| UUA(L) | 329 | 3.73 | UCA(S) | 97 | 2.14 | UAA(*) | 0 | 0 | UGA(W) | 78 | 1.63 |
| UUG(L) | 64 | 0.73 | UCG(S) | 6 | 0.13 | UAG(*) | 0 | 0 | UGG(W) | 18 | 0.38 |
| CUU(L) | 51 | 0.58 | CCU(P) | 56 | 1.68 | CAU(H) | 51 | 1.4 | CGU(R) | 16 | 1.25 |
| CUC(L) | 11 | 0.12 | CCC(P) | 21 | 0.63 | CAC(H) | 22 | 0.6 | CGC(R) | 0 | 0 |
| CUA(L) | 63 | 0.71 | CCA(P) | 54 | 1.62 | CAA(Q) | 52 | 1.73 | CGA(R) | 30 | 2.35 |
| CUG(L) | 11 | 0.12 | CCG(P) | 2 | 0.06 | CAG(Q) | 8 | 0.27 | CGG(R) | 5 | 0.39 |
| AUU(I) | 296 | 1.55 | ACU(T) | 71 | 1.53 | AAU(N) | 145 | 1.46 | AGU(S) | 31 | 0.68 |
| AUC(I) | 87 | 0.45 | ACC(T) | 44 | 0.95 | AAC(N) | 53 | 0.54 | AGC(S) | 12 | 0.26 |
| AUA(M) | 242 | 1.59 | ACA(T) | 69 | 1.48 | AAA(K) | 90 | 1.65 | AGA(S) | 65 | 1.43 |
| AUG(M) | 63 | 0.41 | ACG(T) | 2 | 0.04 | AAG(K) | 19 | 0.35 | AGG(S) | 11 | 0.24 |
| GUU(V) | 56 | 1.46 | GCU(A) | 56 | 1.64 | GAU(D) | 56 | 1.58 | GGU(G) | 73 | 1.39 |
| GUC(V) | 10 | 0.26 | GCC(A) | 30 | 0.88 | GAC(D) | 15 | 0.42 | GGC(G) | 17 | 0.32 |
| GUA(V) | 68 | 1.78 | GCA(A) | 46 | 1.34 | GAA(E) | 66 | 1.67 | GGA(G) | 78 | 1.49 |
| GUG(V) | 19 | 0.5 | GCG(A) | 5 | 0.15 | GAG(E) | 13 | 0.33 | GGG(G) | 42 | 0.8 |

**Table S15** Relative synonymous codon usage (RSCU) of *E. oculatus* mitogenome.

| Codon | Count | RSCU | Codon | Count | RSCU | Codon | Count | RSCU | Codon | Count | RSCU |
| --- | --- | --- | --- | --- | --- | --- | --- | --- | --- | --- | --- |
| UUU(F) | 266 | 1.69 | UCU(S) | 106 | 2.34 | UAU(Y) | 157 | 1.67 | UGU(C) | 42 | 1.83 |
| UUC(F) | 49 | 0.31 | UCC(S) | 27 | 0.6 | UAC(Y) | 31 | 0.33 | UGC(C) | 4 | 0.17 |
| UUA(L) | 348 | 3.86 | UCA(S) | 98 | 2.17 | UAA(*) | 0 | 0 | UGA(W) | 87 | 1.76 |
| UUG(L) | 59 | 0.65 | UCG(S) | 5 | 0.11 | UAG(*) | 0 | 0 | UGG(W) | 12 | 0.24 |
| CUU(L) | 41 | 0.45 | CCU(P) | 63 | 1.94 | CAU(H) | 54 | 1.52 | CGU(R) | 22 | 1.73 |
| CUC(L) | 11 | 0.12 | CCC(P) | 19 | 0.58 | CAC(H) | 17 | 0.48 | CGC(R) | 1 | 0.08 |
| CUA(L) | 78 | 0.87 | CCA(P) | 44 | 1.35 | CAA(Q) | 63 | 1.8 | CGA(R) | 22 | 1.73 |
| CUG(L) | 4 | 0.04 | CCG(P) | 4 | 0.12 | CAG(Q) | 7 | 0.2 | CGG(R) | 6 | 0.47 |
| AUU(I) | 341 | 1.67 | ACU(T) | 70 | 1.63 | AAU(N) | 178 | 1.63 | AGU(S) | 33 | 0.73 |
| AUC(I) | 67 | 0.33 | ACC(T) | 27 | 0.63 | AAC(N) | 40 | 0.37 | AGC(S) | 12 | 0.27 |
| AUA(M) | 267 | 1.74 | ACA(T) | 72 | 1.67 | AAA(K) | 83 | 1.75 | AGA(S) | 71 | 1.57 |
| AUG(M) | 40 | 0.26 | ACG(T) | 3 | 0.07 | AAG(K) | 12 | 0.25 | AGG(S) | 10 | 0.22 |
| GUU(V) | 47 | 1.37 | GCU(A) | 60 | 1.76 | GAU(D) | 53 | 1.56 | GGU(G) | 68 | 1.36 |
| GUC(V) | 11 | 0.32 | GCC(A) | 17 | 0.5 | GAC(D) | 15 | 0.44 | GGC(G) | 16 | 0.32 |
| GUA(V) | 60 | 1.75 | GCA(A) | 49 | 1.44 | GAA(E) | 66 | 1.69 | GGA(G) | 77 | 1.54 |
| GUG(V) | 19 | 0.55 | GCG(A) | 10 | 0.29 | GAG(E) | 12 | 0.31 | GGG(G) | 39 | 0.78 |

**Table S16** Relative synonymous codon usage (RSCU) of *T. nodulosa* mitogenome.

| Codon | Count | RSCU | Codon | Count | RSCU | Codon | Count | RSCU | Codon | Count | RSCU |
| --- | --- | --- | --- | --- | --- | --- | --- | --- | --- | --- | --- |
| UUU(F) | 250 | 1.6 | UCU(S) | 97 | 1.98 | UAU(Y) | 135 | 1.51 | UGU(C) | 33 | 1.61 |
| UUC(F) | 62 | 0.4 | UCC(S) | 37 | 0.76 | UAC(Y) | 44 | 0.49 | UGC(C) | 8 | 0.39 |
| UUA(L) | 298 | 3.25 | UCA(S) | 102 | 2.08 | UAA(*) | 0 | 0 | UGA(W) | 69 | 1.47 |
| UUG(L) | 83 | 0.9 | UCG(S) | 16 | 0.33 | UAG(*) | 0 | 0 | UGG(W) | 25 | 0.53 |
| CUU(L) | 53 | 0.58 | CCU(P) | 56 | 1.68 | CAU(H) | 45 | 1.23 | CGU(R) | 19 | 1.52 |
| CUC(L) | 20 | 0.22 | CCC(P) | 31 | 0.93 | CAC(H) | 28 | 0.77 | CGC(R) | 4 | 0.32 |
| CUA(L) | 81 | 0.88 | CCA(P) | 40 | 1.2 | CAA(Q) | 55 | 1.57 | CGA(R) | 20 | 1.6 |
| CUG(L) | 16 | 0.17 | CCG(P) | 6 | 0.18 | CAG(Q) | 15 | 0.43 | CGG(R) | 7 | 0.56 |
| AUU(I) | 262 | 1.42 | ACU(T) | 68 | 1.51 | AAU(N) | 135 | 1.47 | AGU(S) | 46 | 0.94 |
| AUC(I) | 106 | 0.58 | ACC(T) | 39 | 0.87 | AAC(N) | 49 | 0.53 | AGC(S) | 11 | 0.22 |
| AUA(M) | 234 | 1.61 | ACA(T) | 70 | 1.56 | AAA(K) | 81 | 1.54 | AGA(S) | 80 | 1.63 |
| AUG(M) | 56 | 0.39 | ACG(T) | 3 | 0.07 | AAG(K) | 24 | 0.46 | AGG(S) | 3 | 0.06 |
| GUU(V) | 73 | 1.77 | GCU(A) | 56 | 1.59 | GAU(D) | 52 | 1.42 | GGU(G) | 68 | 1.26 |
| GUC(V) | 11 | 0.27 | GCC(A) | 32 | 0.91 | GAC(D) | 21 | 0.58 | GGC(G) | 19 | 0.35 |
| GUA(V) | 62 | 1.5 | GCA(A) | 47 | 1.33 | GAA(E) | 60 | 1.62 | GGA(G) | 83 | 1.54 |
| GUG(V) | 19 | 0.46 | GCG(A) | 6 | 0.17 | GAG(E) | 14 | 0.38 | GGG(G) | 46 | 0.85 |

**Table S17** Relative synonymous codon usage (RSCU) of *S. melli* mitogenome.

| Codon | Count | RSCU | Codon | Count | RSCU | Codon | Count | RSCU | Codon | Count | RSCU |
| --- | --- | --- | --- | --- | --- | --- | --- | --- | --- | --- | --- |
| UUU(F) | 211 | 1.36 | UCU(S) | 98 | 2.07 | UAU(Y) | 111 | 1.35 | UGU(C) | 36 | 1.36 |
| UUC(F) | 99 | 0.64 | UCC(S) | 27 | 0.57 | UAC(Y) | 54 | 0.65 | UGC(C) | 17 | 0.64 |
| UUA(L) | 227 | 2.53 | UCA(S) | 96 | 2.03 | UAA(*) | 0 | 0 | UGA(W) | 65 | 1.29 |
| UUG(L) | 86 | 0.96 | UCG(S) | 10 | 0.21 | UAG(*) | 0 | 0 | UGG(W) | 36 | 0.71 |
| CUU(L) | 67 | 0.75 | CCU(P) | 65 | 1.86 | CAU(H) | 38 | 1.07 | CGU(R) | 19 | 1.49 |
| CUC(L) | 31 | 0.35 | CCC(P) | 31 | 0.89 | CAC(H) | 33 | 0.93 | CGC(R) | 3 | 0.24 |
| CUA(L) | 95 | 1.06 | CCA(P) | 35 | 1 | CAA(Q) | 56 | 1.58 | CGA(R) | 24 | 1.88 |
| CUG(L) | 33 | 0.37 | CCG(P) | 9 | 0.26 | CAG(Q) | 15 | 0.42 | CGG(R) | 5 | 0.39 |
| AUU(I) | 213 | 1.37 | ACU(T) | 67 | 1.24 | AAU(N) | 101 | 1.24 | AGU(S) | 44 | 0.93 |
| AUC(I) | 99 | 0.63 | ACC(T) | 53 | 0.98 | AAC(N) | 62 | 0.76 | AGC(S) | 18 | 0.38 |
| AUA(M) | 213 | 1.48 | ACA(T) | 88 | 1.62 | AAA(K) | 82 | 1.67 | AGA(S) | 70 | 1.48 |
| AUG(M) | 75 | 0.52 | ACG(T) | 9 | 0.17 | AAG(K) | 16 | 0.33 | AGG(S) | 16 | 0.34 |
| GUU(V) | 76 | 1.47 | GCU(A) | 57 | 1.42 | GAU(D) | 48 | 1.28 | GGU(G) | 61 | 1.1 |
| GUC(V) | 23 | 0.44 | GCC(A) | 41 | 1.02 | GAC(D) | 27 | 0.72 | GGC(G) | 31 | 0.56 |
| GUA(V) | 74 | 1.43 | GCA(A) | 51 | 1.27 | GAA(E) | 53 | 1.36 | GGA(G) | 71 | 1.29 |
| GUG(V) | 34 | 0.66 | GCG(A) | 12 | 0.3 | GAG(E) | 25 | 0.64 | GGG(G) | 58 | 1.05 |

**Table S18** Relative synonymous codon usage (RSCU) of *P. sichuanense* mitogenome.

| Codon | Count | RSCU | Codon | Count | RSCU | Codon | Count | RSCU | Codon | Count | RSCU |
| --- | --- | --- | --- | --- | --- | --- | --- | --- | --- | --- | --- |
| UUU(F) | 260 | 1.59 | UCU(S) | 110 | 2.46 | UAU(Y) | 151 | 1.62 | UGU(C) | 45 | 1.61 |
| UUC(F) | 67 | 0.41 | UCC(S) | 29 | 0.65 | UAC(Y) | 35 | 0.38 | UGC(C) | 11 | 0.39 |
| UUA(L) | 288 | 3.37 | UCA(S) | 98 | 2.2 | UAA(*) | 0 | 0 | UGA(W) | 76 | 1.57 |
| UUG(L) | 75 | 0.88 | UCG(S) | 7 | 0.16 | UAG(*) | 0 | 0 | UGG(W) | 21 | 0.43 |
| CUU(L) | 67 | 0.78 | CCU(P) | 56 | 1.68 | CAU(H) | 55 | 1.55 | CGU(R) | 21 | 1.5 |
| CUC(L) | 13 | 0.15 | CCC(P) | 24 | 0.72 | CAC(H) | 16 | 0.45 | CGC(R) | 6 | 0.43 |
| CUA(L) | 53 | 0.62 | CCA(P) | 47 | 1.41 | CAA(Q) | 69 | 1.86 | CGA(R) | 25 | 1.79 |
| CUG(L) | 17 | 0.2 | CCG(P) | 6 | 0.18 | CAG(Q) | 5 | 0.14 | CGG(R) | 4 | 0.29 |
| AUU(I) | 290 | 1.58 | ACU(T) | 71 | 1.49 | AAU(N) | 129 | 1.52 | AGU(S) | 31 | 0.69 |
| AUC(I) | 78 | 0.42 | ACC(T) | 26 | 0.55 | AAC(N) | 41 | 0.48 | AGC(S) | 9 | 0.2 |
| AUA(M) | 238 | 1.6 | ACA(T) | 83 | 1.75 | AAA(K) | 89 | 1.66 | AGA(S) | 66 | 1.48 |
| AUG(M) | 59 | 0.4 | ACG(T) | 10 | 0.21 | AAG(K) | 18 | 0.34 | AGG(S) | 7 | 0.16 |
| GUU(V) | 78 | 1.71 | GCU(A) | 61 | 1.63 | GAU(D) | 55 | 1.59 | GGU(G) | 68 | 1.28 |
| GUC(V) | 6 | 0.13 | GCC(A) | 33 | 0.88 | GAC(D) | 14 | 0.41 | GGC(G) | 23 | 0.43 |
| GUA(V) | 73 | 1.6 | GCA(A) | 48 | 1.28 | GAA(E) | 60 | 1.48 | GGA(G) | 74 | 1.39 |
| GUG(V) | 25 | 0.55 | GCG(A) | 8 | 0.21 | GAG(E) | 21 | 0.52 | GGG(G) | 48 | 0.9 |
